# Specific Interaction between eEF1A and HIV RT Is Critical for HIV-1 Reverse Transcription and a Potential Anti-HIV Target

**DOI:** 10.1371/journal.ppat.1005289

**Published:** 2015-12-01

**Authors:** Dongsheng Li, Ting Wei, Daniel J. Rawle, Fangyun Qin, Rui Wang, Dinesh C. Soares, Hongping Jin, Haran Sivakumaran, Min-Hsuan Lin, Kirsten Spann, Catherine M. Abbott, David Harrich

**Affiliations:** 1 Department of Cell and Molecular Biology, QIMR Berghofer Medical Research Institute, Herston, Queensland, Australia; 2 School of Chemistry and Molecular Biosciences, University of Queensland, St. Lucia, Queensland, Australia; 3 Medical Genetics Section, Centre for Genomic and Experimental Medicine, MRC Institute of Genetics and Molecular Medicine, Western General Hospital, University of Edinburgh, Edinburgh, United Kingdom; 4 School of Biomedical Science, Queensland University of Technology, Brisbane, Queensland, Australia; University of Wisconsin, UNITED STATES

## Abstract

Reverse transcription is the central defining feature of HIV-1 replication. We previously reported that the cellular eukaryotic elongation factor 1 (eEF1) complex associates with the HIV-1 reverse transcription complex (RTC) and the association is important for late steps of reverse transcription. Here we show that association between the eEF1 and RTC complexes occurs by a strong and direct interaction between the subunit eEF1A and reverse transcriptase (RT). Using biolayer interferometry and co-immunoprecipitation (co-IP) assays, we show that association between the eEF1 and RTC complexes occurs by a strong (K_D_ ~3–4 nM) and direct interaction between eEF1A and reverse transcriptase (RT). Biolayer interferometry analysis of cell lysates with titrated levels of eEF1A indicates it is a predominant cellular RT binding protein. Both the RT thumb and connection domains are required for interaction with eEF1A. A single amino acid mutation, W252A, within the thumb domain impaired co-IP between eEF1A and RT, and also significantly reduced the efficiency of late reverse transcription and virus replication when incorporated into infectious HIV-1. Molecular modeling analysis indicated that interaction between W252 and L303 are important for RT structure, and their mutation to alanine did not impair heterodimerisation, but negatively impacted interaction with eEF1A. Didemnin B, which specifically binds eEF1A, potently inhibited HIV-1 reverse transcription by greater than 2 logs at subnanomolar concentrations, especially affecting reverse transcription late DNA synthesis. Analysis showed reduced levels of RTCs from HIV-1-infected HEK293T treated with didemnin B compared to untreated cells. Interestingly, HIV-1 with a W252A RT mutation was resistant to didemnin B negative effects showing that didemnin B affects HIV-1 by targeting the RT-eEF1A interaction. The combined evidence indicates a direct interaction between eEF1A and RT is crucial for HIV reverse transcription and replication, and the RT-eEF1A interaction is a potential drug target.

## Author Summary

After infecting a target cell, HIV-1 like all retroviruses converts the viral single strand RNA genome into double strand DNA by the process called reverse transcription. Host proteins are known to be important for reverse transcription yet a direct role for any host protein has not been demonstrated. In this paper, we show that a eukaryotic translation elongation factor (eEF1A), an abundant cellular protein, directly and strongly binds to the viral enzyme reverse transcriptase (RT). The biological relevance of the association is supported by mutational analysis of RT and by treating cells with the small molecule didemnin B that specifically binds eEF1A. Mutation of RT or treatment of cells with didemnin B resulted in significantly decreased efficiency of reverse transcription. Didemnin B treatment of cells disrupted HIV-1’s ability to maintain the viral machinery necessary for reverse transcription. However, an HIV-1 mutant, which does not interact with eEF1A, was resistant to didemnin B negative effects on early viral replication, showing that didemnin B affects HIV-1 by targeting the RT-eEF1A interaction. Altogether, this study demonstrates that eEF1A is an integral component of the viral reverse transcription complex and that the RT-eEF1A interaction is a possible new drug target to inhibit HIV-1 replication.

## Introduction

To convert the viral genomic RNA into double stranded DNA, HIV-1 forms a prototypical ribonucleoprotein complex on the viral genomic RNA called the reverse transcription complex (RTC) [1]. It includes the viral enzymes reverse transcriptase (RT) and integrase (IN), as well as other viral proteins including capsid (CA), matrix (MA), viral protein R (Vpr), and viral infectivity factor (Vif). The complex assembles on the 5’ untranslated region (UTR) of HIV-1 genomic RNA and requires tRNA^Lys3^, which anneals to a viral primer binding site sequence so that DNA synthesis can initiate. Reverse transcription can also be regulated by viral proteins such as Tat [2–5]. HIV-1 reverse transcription begins shortly after the viral core enters the cell cytoplasm, when nucleotides become available to make a short single strand of DNA referred to as minus strand strong stop DNA (sssDNA or early DNA). Studies show that completion of *in vitro* reverse transcription can be greatly enhanced by the addition of cell lysate, indicating that one or more cellular activities are required to form RTC and enable its full function [6–8].

Recently, two subunits of the eukaryotic elongation factor 1 (eEF1) complex, eEF1A1 (hereafter referred to as eEF1A) and eEF1G, were found to associate with RT and IN [9]. The cellular eEF1 complex is composed of several subunit proteins and co-factors [10]. These include eEF1A and the eEF1B complex (composed of eEF1G, eEF1B, eEF1D and valyl-tRNA synthetase). During protein synthesis, eEF1A binds to and delivers aminoacetylated transfer RNAs (aa-tRNAs) to the elongating ribosome. In the study by Warren et al. [9], only cell lysate fractions containing the eEF1 complex strongly stimulated late stages of reverse transcription. In HIV-1 infected cells, eEF1 components eEF1A and eEF1G were shown to co-purify with the RTC and depletion of either by siRNA knock down resulted in instability of the RTC in cells, which reduces synthesis of late DNA but not early DNA products of reverse transcription [9]. These outcomes were consistent with the previous observation that cell lysate stimulated late steps of reverse transcription [6,11]. Exactly how eEF1 subunits interact with the RTC is unknown, and therefore the focus of this study.

This study aimed to characterize the association between eEF1 and HIV-1 RTC complexes, and its roles in virus reverse transcription and replication. We found that a specific, strong and direct binding between eEF1A and RT is a dominant interaction that can account for the association between eEF1 and RTC complexes.

## Results

### eEF1A exhibits strong and direct binding to HIV-1 RT

**Fig 1:**
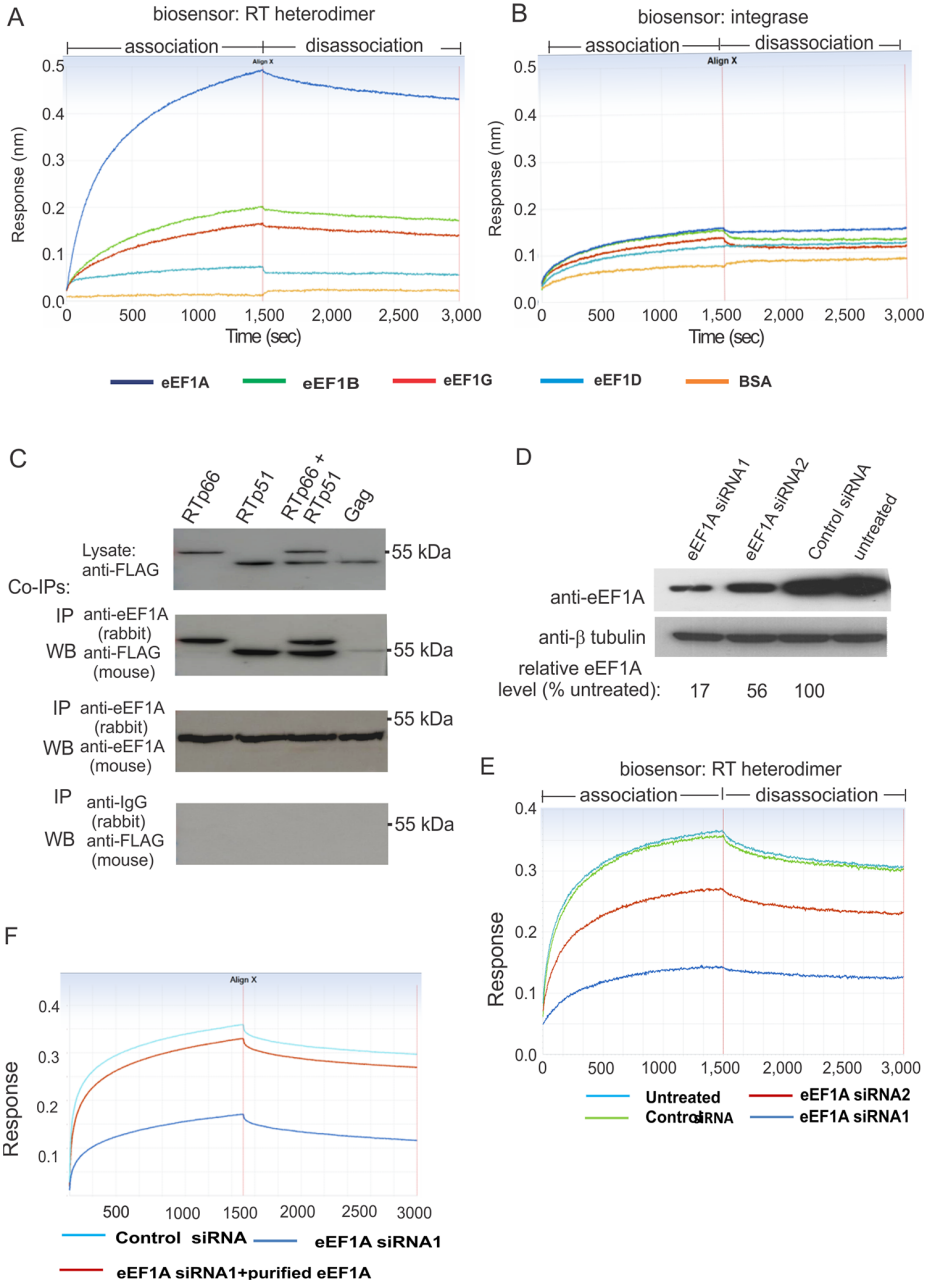
eEF1A binds directly and tightly to HIV-1 RT *in vitro*. (**A**) Biolayer interferometry assays (BLI) of a biotinylated HIV RTp66/p51-loaded biosensor with 60 nM of purified eEF1A, eEF1B, eEF1D, eEF1G or BSA as analytes. (**B**) BLI assays using a biotinylated HIV-1 IN-loaded biosensor as described in (**A**). (**C**) Co-IP of RT with eEF1A. HEK 293T cells were transfected with plasmids to express FLAG-tagged RTp66, RTp51, both RTp51 and RTp66, or Gag. Expression of the proteins in cell lysates was confirmed by western blot using anti-FLAG antibody (upper panel). The cell lysate was used for co-IP analysis with a rabbit anti-eEF1A capture antibody. Capture of RT subunits and Gag was examined by western blot with an anti-FLAG antibody (second panel). Western blot with a mouse anti-eEF1A antibody was used to confirm eEF1A capture (third panel). Nonspecific binding to the beads was excluded by IP using an anti-rabbit IgG capture antibody followed by western blot using an anti-FLAG antibody (lower panel). (**D**) HEK293T cells were treated with two eEF1A siRNAs, one control siRNA or no siRNA for 48 h before preparing a cell lysate. The levels of eEF1A (upper panel) and β tubulin (lower panel) in the cell lysate were determined by western blot. Densitometry analysis of the western blot image was used to calculate the relative levels of eEF1A in each siRNA-treated sample compared to the untreated control (**E**) Sensorgrams of association and dissociation of cell lysates containing various levels of eEF1A to RTp66-biosensors. (**F**) Addition of purified MYC/DDK-eEF1A1 protein to a cell lysate where eEF1A was strongly reduced by siRNA treatment restored a sensograms profile indicative of strong RT interaction. All experiments were performed at least three times and representative results are shown.

To further characterize the association between eEF1 complex and the RTC, we used biolayer interferometry (BLI) assays to determine if a specific subunit of the eEF1 complex could directly bind to RT or IN. The BLI method uses the interference pattern of white light reflected from two surfaces on a biosensor probe: a layer of immobilized protein of interest and an internal reference layer. Any change in the number of molecules bound to the biosensor tip due to protein-protein interaction causes a shift in the interference pattern that can be measured. Purified RT p66/p51 heterodimer was biotinylated and immobilized on a streptavidin-coated Octet-Red system biosensor. The interaction of RT p66/51 with individual subunits of the eEF1 complex, including eEF1A, eEF1B, eEF1G and eEF1D, was assessed using solutions containing 60 nM of individual recombinant eEF1 subunits. Only eEF1A demonstrated strong binding with RT, while weak binding was observed between eEF1B and eEF1G with RT and no interaction was detected between eEF1D and RT under the conditions tested (Fig 1A). A similar experiment was performed using a biosensor labeled with HIV-1 IN and the same eEF1 subunits. All subunits of eEF1 showed very weak or negligible interaction with IN (Fig 1B). We performed a reciprocal experiment in which biosensors were labeled with individual eEF1 subunits and incubated with solutions containing RT heterodimer at 90, 30 and 10 nM. A concentration-dependent binding of eEF1A with RT was detected along with weak binding of eEF1B (S1 Fig). These results indicate that an association of the HIV-1 RTC with the eEF1complex, as identified in our early study [9], is occurring via the direct binding between RT and eEF1A components of the two complexes, respectively.

Next we compared the interaction of individual RT subunits or the heterodimer with eEF1A using eEF1A-labeled biosensors. HIV-1 Gag protein, a previously reported eEF1A binding protein [12] was included as a control. The BLI assays showed a concentration-dependent interaction between the proteins tested (S2 Fig). The goodness of curve fit was indicated by low apparent χ^2^ of 1.07 to 2.12, where below 10 is considered acceptable [13]. The combined results revealed a binding hierarchy where RTp66 ≈ RTp66/p51 > RTp51 > Gag. The K_D_ of eEF1A with RT subunits ranged from 3–4 nM for RTp66, RTp66/p51 and RTp51 and 21 nM for Gag under the conditions tested, indicating that each RT subunit can form a stable protein complex with eEF1A (Table 1).

**Table 1:** Association and dissociation parameters of eEF1A with RT subunits, RT heterodimer and Gag in BLI assays.

	Ka (1/MS) ^(1)^	Kd (1/second) ^(2)^	K_D_ (nM) ^(3)^	χ^2^ ^(4)^
RT p51	4.48 ± 2.31×10^4^	1.85 ± 3.67×10^−4^	4.13 ± 2.32	1.25 ± 0.46
RT p66	6.95 ± 4.68×10^4^	2.22 ± 1.05×10^−4^	3.19 ± 1.08	2.12 ± 1.08
RT p51/66	5.52 ± 3.16×10^4^	2.07 ± 1.72×10^−4^	3.75 ± 1.04	1.86 ± 0.78
Gag	8.75 ± 4.43×10^4^	1.88 ± 2.56×10^−3^	21.49 ± 11.08	1.07 ± 0.29

^(1)^ association rate constants (k_a_): The rate of complex formed per second in a 1 molar solution of each component.

^(2)^ dissociation rate constant (k_d_): Stability of the complex, i.e. the fraction of complexes decays per second.

^(3)^ affinity constant (K_D_): K_D_ = k_d_/k_a_.

^(4)^ chi-square test

The binding of RT subunits and Gag with eEF1A was confirmed using co-immunoprecipitation (co-IP) experiments. RT subunits and Gag, each with a C-terminal FLAG-tag, were expressed in HEK293T cells individually or in combination (Fig 1C). Cell lysates prepared 24 h post-transfection were incubated with protein G-conjugated agarose beads bound with a rabbit anti-eEF1A antibody. Western blot analysis of immunoprecipitated proteins indicated that RT subunits associated with eEF1A better than Gag (Fig 1C). A reciprocal IP experiment showed that RTp51 could co-IP eEF1A (S3 Fig). Our combined data indicates that eEF1A interacts with individual RT subunits or the RT heterodimer both directly and strongly.

As eEF1A is an abundant cellular protein that tightly binds HIV-1 RT, it is possible that eEF1A is a dominant cellular RT binding protein. To examine this possibility, eEF1A levels were downregulated using previously described siRNAs [9] and the association of total cellular protein with an RTp66/p51 biosensor was examined by BLI. In BLI only molecules binding to or dissociating from the biosensor can shift the interference pattern. Hence crude cell lysate can be used in BLI assays to investigate protein-protein interaction [14]. Protein concentration of siRNA-treated and control lysates was carefully monitored using a bioanalyzer and confirmed by western blot using an anti-β tubulin antibody (Fig 1D, bottom panel). The eEF1A siRNA1 and siRNA2 treated cell lysates had reduced eEF1A levels of >80% and >40%, respectively, and showed reduced association with the RT biosensor compared to lysates from control siRNA treated cells and untreated cell lysates (Fig 1E), which correlated to the levels of eEF1A present in each cell lysate (Fig 1D, top panel). Addition of purified MYC/DDK-tagged eEF1A to an eEF1A-depleted cell lysate restored the BLI interference response (Fig 1F) showing that the response curves reflected eEF1A levels in the cell lysates. The results provide evidence that eEF1A is a predominant RT binding protein in cells.

### The RT thumb and connection domains are important for interaction with eEF1A

**Fig 2:**
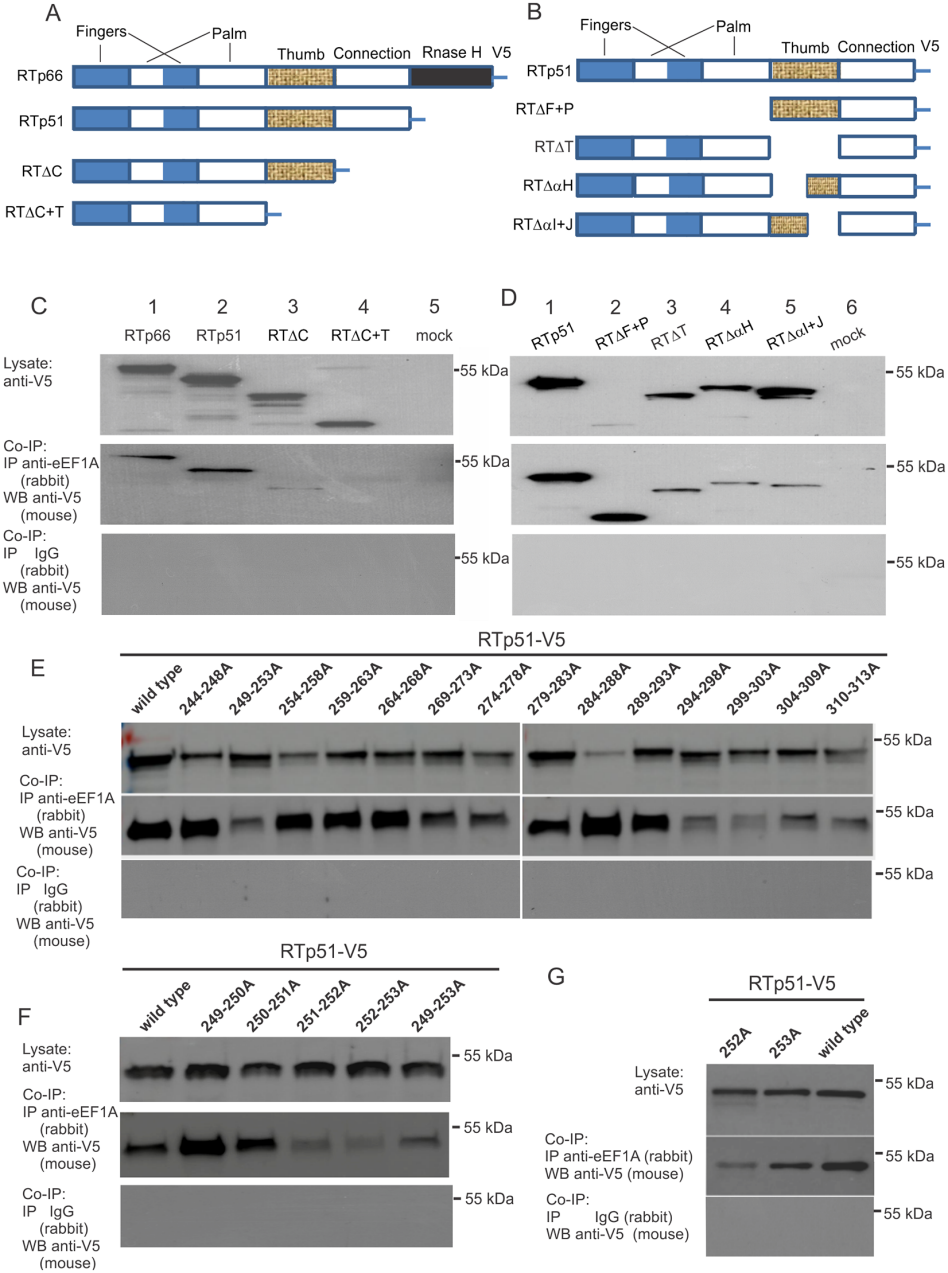
The RT thumb and connection domains are required for RT-eEF1A interaction. (**A** and **B**) Schematic diagrams representing V5-tagged RT truncation and deletion mutants, indicating important RT domains. (**C** and **D**) Lysates from cells transiently expressing each wild type or mutant RT subunit, as indicated (top panels), were immunoprecipitated with an anti-eEF1A antibody and analyzed by western blot with an anti-V5 antibody (middle panels). Nonspecific binding to the beads was excluded by IP using an anti-rabbit IgG capture antibody followed by western blot using an anti-V5 antibody (bottom panels). (**E**) Lysates from transiently transfected cells expressing RTp51-V5 having non-overlapping mutations consisting of 5 consecutive alanine substitutions, (**F**) overlapping double alanine substitutions), or (**G**) single alanine substitutions (top panels) were visualized by western blot. Co-IP of RTp51 and RTp66 proteins using an anti-eEF1A antibody were visualized by western blot with an anti-V5 antibody (middle panels). Nonspecific binding to the beads was excluded by IP using an anti-rabbit IgG capture antibody followed by western blot using an anti-V5 antibody (bottom panels). All experiments were performed at least three times and representative results are shown.

To determine the region of RT that is important for interaction with eEF1A, a series of C-terminal deletions of RT domains were made, each tagged with a C-terminal V5 epitope (Fig 2A). The wild type or mutated RT expression plasmids were transfected into HEK293T cells and western blots were performed to show that all RT proteins were expressed (Fig 2C, top panel). Immunoprecipitation of eEF1A showed reduced co-IP of RT when the connection domain was deleted, and no co-IP was detected when both the thumb and connection domains were deleted (Fig 2C, middle panel). The results indicate that the RT thumb and connection domains are required for interaction with eEF1A. Another series of N-terminal and internal deletions in RT were made to further investigate the role played by the thumb and connection domains in the eEF1A interaction (Fig 2B). An expression plasmid containing only the thumb and connection domains (RT-ΔF+P) expressed protein at low levels (Fig 2D, top panel) but was strongly co-immunoprecipitated by eEF1A (Fig 2D, middle panel), confirming that the thumb and connection domains are important for binding to eEF1A. The RT thumb domain (residues 244–323) contains 3 alpha helices: helix H (residue 255–268), helix I (residues 278–286) and helix J (residues 298–311). Three RT constructs having internal deletions in the thumb domain were made to see if the entire thumb domain or a specific region was required for interaction with eEF1A. Deletion of the whole thumb domain, deletion of helix H alone and deletion of helices I and J combined, each resulted in a reduced co-IP of RT with eEF1A (Fig 2D). The combined data show that the connection and thumb domains of RT are required for interaction with eEF1A where multiple binding determinants in these domains mediate a stable protein interaction with eEF1A.

### Alanine substitution mutagenesis reveals residues in the thumb domain critical for RT interaction with eEF1A

As deletion of either N- or C-terminal regions of the RT thumb domain impaired the RT-eEF1A interaction, we used alanine substitution mutations to screen positions in the thumb domain that could mediate the RT-eEF1A interaction. We mutated the RTp51-V5 expression plasmid to make 15 non-overlapping substitutions consisting of 5 consecutive alanine residues that covered the thumb domain of RT. The RT expression plasmids were individually transfected into HEK293T cells and western blots of cell lysates prepared from the cells showed that each RT protein was made (Fig 2E, top panels). Co-IP analysis using an anti-eEF1A antibody identified mutations in two regions of the thumb domain, residues 249–253 and 294–313, that downregulated association of RT p51 with eEF1A (Fig 2E, middle panels). The mutation of residues 249–253 was investigated further by introducing overlapping double alanine mutations to make four new mutant RTp51-V5 expression plasmids: 249-250A, 250-251A, 251-252A and 252-253A. Co-IP experiments showed that the alanine substitutions 251-252A and 252-253A impaired interaction with eEF1A (Fig 2F), suggesting that W252 is important for the interaction. Single alanine substitutions at residues W252 or T253 were further evaluated and the W252A mutation significantly inhibited co-IP of RT with eEF1A, while the effect of the T253A mutation was minor compared to co-IP of wild type RT (Fig 2G). The result indicates that maintenance of W252 in RT is particularly important for its interaction with eEF1A.

### The W252A mutation is important for RT structure but does not impair heterodimer formation

**Fig 3:**
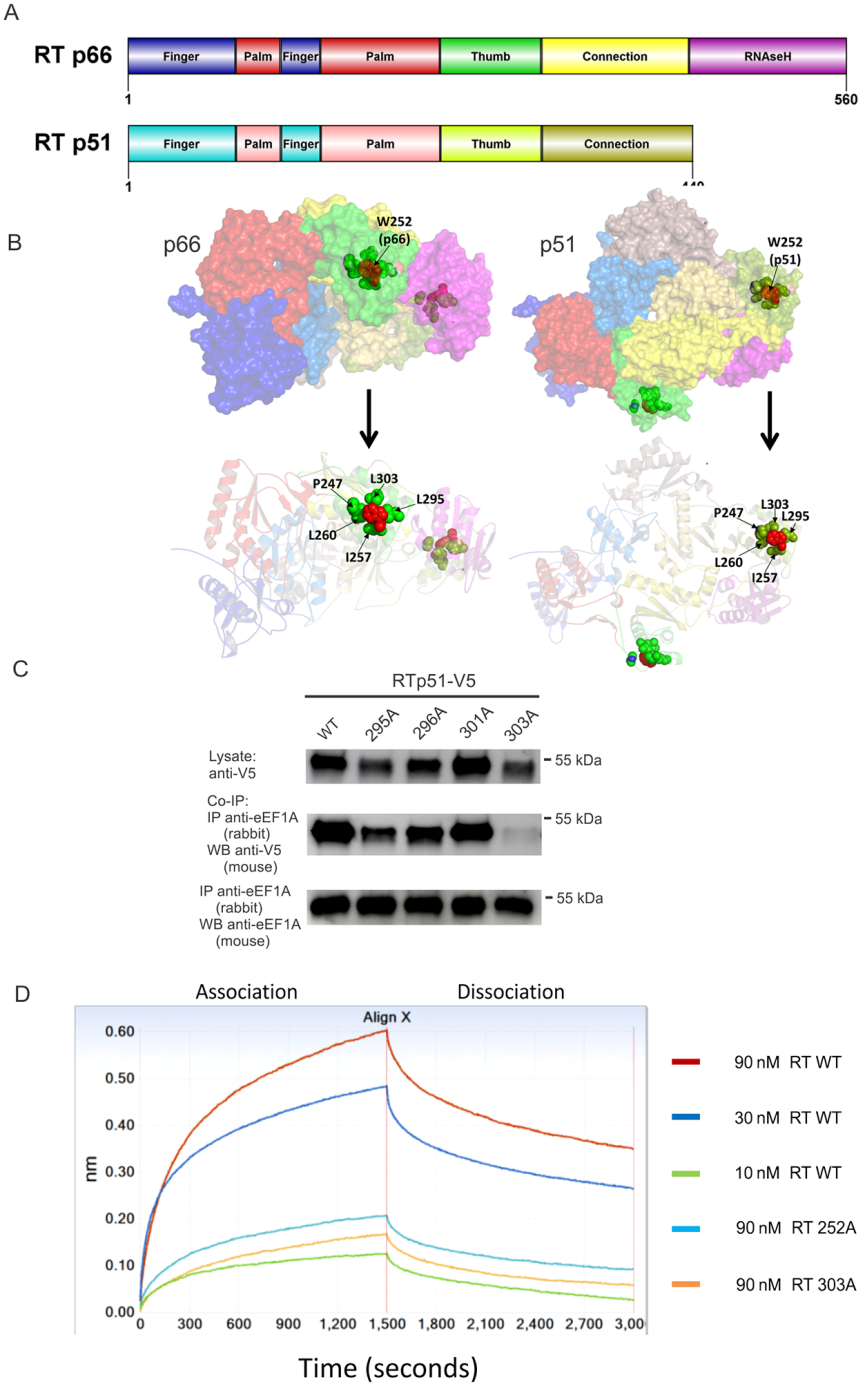
Point mutations in the thumb domain affect RT structure and interaction with eEF1A. (**A**) Schematic diagrams representing domains of RT p66 and p51. (**B**) Surface representation of the p66/p51 heterodimer is shown along with a ribbon schematic representation, colored by domain with a transparency setting to allow visualization of the buried W252 residue and its interacting residues. The location of the W252 residue is shown; its side-chain atoms are shown as red spheres. Residues that make hydrophobic contacts with W252 are shown colored green and labeled. The side-chain of W252 supports numerous hydrophobic interactions with spatially proximal residues, including P247, I257, L260, L295 and L303. (**C**) Co-IP assays were performed with wild type and RT mutations predicted to interact with RT W252. Mutations flanking L295A and L303A, T296A and L301A, were included as controls. Only the L303A RT mutation reduced interaction with eEF1A in a co-IP assay as performed in Fig 2. The experiment was performed three times with similar results and a representative data set is shown. (**D**) A BLI assay using purified eEF1A immobilized on the biosensors and purified 6ᵡHis-tagged RTp66 protein in the solution. The association of 90 nM of wild type RT and RT mutant proteins was compared as indicated. The data is presented as a mean value ± standard deviation from 3 independent experiments. *p<0.05.

The X-ray crystal structure of the HIV-RT p66/p51 heterodimer (PDB B ID: 4G1Q, resolution 1.51 Å) [15] is shown (S4 Fig). The individual subunits that make up the heterodimer are identical in sequence, with the exception of the additional domain present in p66, absent in p51 (Fig 3A). The finger and palm structural domains in both subunits are made up from discontinuous regions of sequence. Structurally, the W252A mutation is located within the loop region that links the palm domain to the thumb domain, proximal to the first alpha-helix of the thumb domain, but distal to the inter-subunit interface (S4 Fig). The bulky aromatic side-chain of W252 is completely buried within the core of the protein, and supports numerous hydrophobic interactions with spatially proximal residues, including P247 (also located within the loop region between palm and thumb domains) and other residues in the thumb domain, I257, L260, L295 and L303 in both subunits. These interactions are mirrored in both subunits in this high resolution structure (Fig 3B). Given that W252 side-chain is buried in the RT core, it is unlikely to directly facilitate interaction between RT and eEF1A.

Thermodynamic stability calculations using the empirical forcefield FoldX program show that the mutation W252A severely destabilizes structure in both individual subunits and within the heterodimer (ΔΔG of mutations range between ~4.7–5.3 kcal/mol) (Table 2). In contrast, mutation T253A reveals no adverse effect on protein stability [16–18]. This is in keeping with analysis of the lowest energy conformer of 252A (in both individual subunits and the heterodimer), which shows that critical hydrophobic interactions with three of the aforementioned interacting residues, P247, L260 and L303, are lost in the absence of the aromatic side-chain of W252 and could render this region more “open” or flexible. The predicted reduced stability of W252A could therefore have consequences on protein conformation that could in turn impact on RT:eEF1A interaction. To test this possibility further, wild type RTp51 or p51 with the single mutations L295A, T296A, L301A, and L303A were subjected to co-IP analysis as previously described. These residues were chosen because L295 and L303 interact with W252, and are in the region of 294–303 where mutation significantly impairs binding to eEF1A (Fig 2C), while neighbouring residues T296 and L301 were mutated to alanine for comparison. While the interaction between eEF1A and L295A, T296A and L301A were comparable to wild type RT, the L303A mutant, like W252A, interacted poorly with eEF1A (Fig 3C), which likely accounts for the reduction in interaction of the 299-303A amino acid mutations with eEF1A. These results suggest that a spatially proximal region, supported by W252 and L303 hydrophobic interactions, are necessary for interaction with eEF1A. A reduced interaction of eEF1A with RT W252A and L303A was further confirmed using BLI assay. The eEF1A biosensors were incubated in bacterially produced 6×his recombinant wild type RTp66 at 90, 30 and 10 nM, or mutant RTp66 W252A and L303A RT at 90 nM (Fig 3E; S5A and S5B Fig). The binding responses of 90 nM 252A or 303A with eEF1A was substantially lower than 90 nM wild type RT, and on par with responses measured for 10 nM of wild type RTp66. As L303A and W252A RT mutation have similar affect on interaction with eEF1A, W252A was selected for use in further experiments.

**Table 2:** *In silico* calculation of the effect of HIV-1 RT p66/p51 mutations on protein stability compared to wild type.

Mutation	Stability Energy Calculation (Average ΔΔG, kcal/mol) ^1^
W252A (p51)	5.30
W252A (p66)	4.68
W252A (p51 in p66/p51 heterodimer	5.27
W252A (p66 in p66/p51 heterodimer)	4.71
T253A (p51)	0.37
T253A (p66)	0.62
T253A (p51 in p66/p51 heterodimer)	0.29
T253A (p66 in p66/p51 heterodimer)	0.64

^1^. Average of 3 calculation runs is reported.

RT heterodimer formation requires interaction between the connection domains of RTp51 and RTp66. In order to assess the effect of the W252A mutation on RT heterodimerization, a mammalian protein-protein interaction trap (MAPPIT) assay was undertaken [19,20] (S6 Fig). We co-transfected various combinations of wild type and mutant RT p51 and RT p66 prey and bait expression plasmids along with the reporter and a transfection control plasmid pCMV-Gluc2 in HEK293T cells and compared firefly luciferase levels induced by leptin stimulation (S7 Fig). The W252A and T253A mutations did not affect luciferase expression indicating that heterodimerization between R p51 and RT p66 harboring mutations were not affected. As previously reported, L234A had a significant reduction of the signal indicating this mutation negatively affect RT dimerization thus validating the system [21,22]. The results therefore indicate that W252A imparts only a localized effect on structure stability.

### The W252A mutation regulates reverse transcription *in vitro*

**Fig 4:**
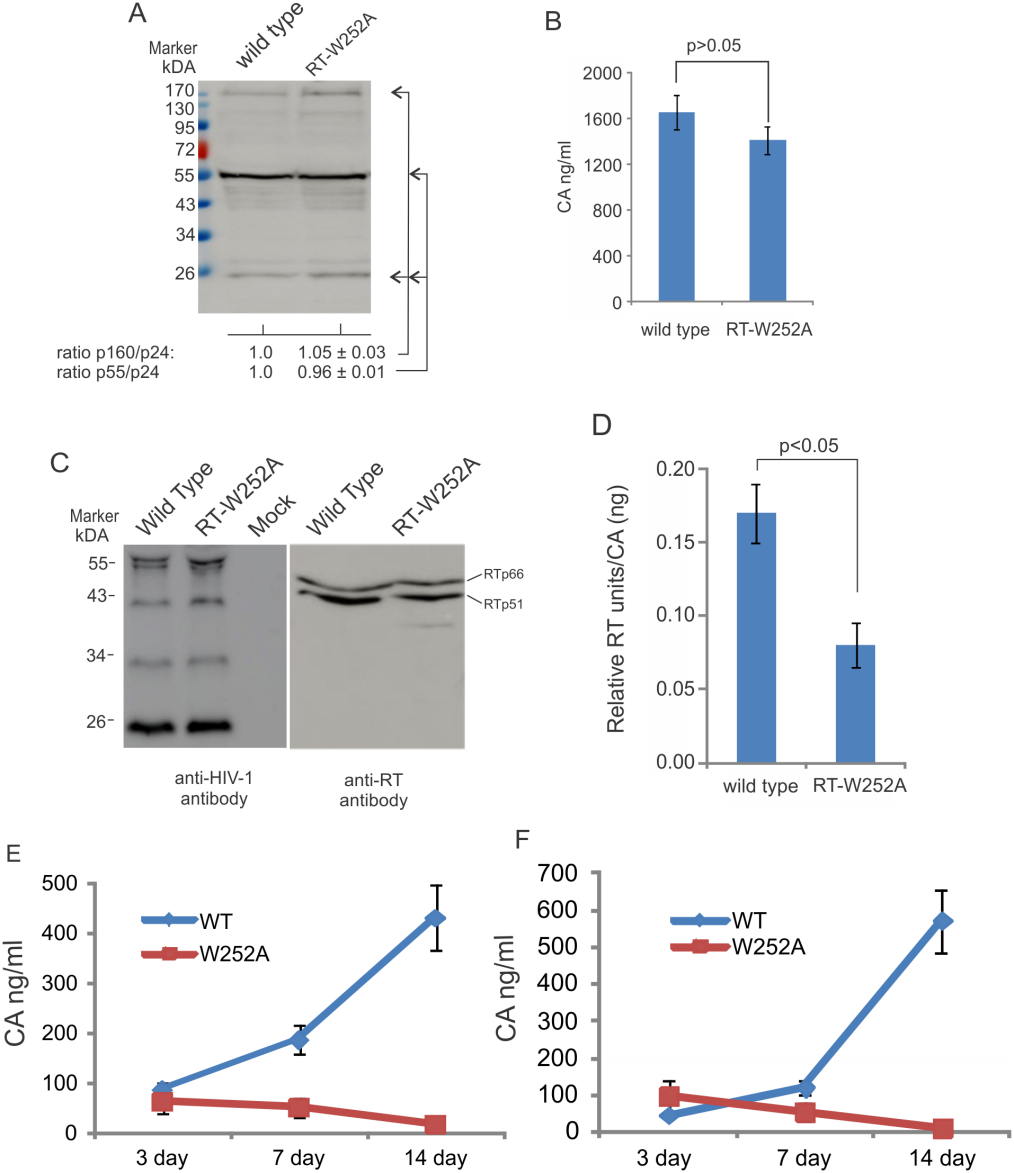
A W252A RT mutation inhibits HIV-1 reverse transcription and replication. (**A**) Proviral plasmids transiently expressing wild type or W252A RT mutant HIV-1 in HEK293T cells were visualized by western blot using a rabbit anit-HIV-1 antibody. Western blot samples were normalized for transfection efficiency using a co-transfected *Gaussia* luciferase reporter plasmid. (**B**) The amount of virus present in culture supernatant was measured using a CA ELISA. Results are normalized for transfection efficiency as determined by secreted *Gaussia* luciferase. (**C**) Western blot of concentrated HIV-1 virus particles from culture supernatants expressing wild type or W252A RT HIV-1, detecting with anti-HIV-1 (left, human serum, NIH AIDS reagent) or anti-RT antibodies (right). (**D**) The RT activities of wild type and W252A RT mutated viruses were measured using a standard RT colorimetric assay and normalized to CA levels in each sample. (**E**) Jurkat cells were infected with wild type or W252A RT mutant virus using equivalent amount of CA (500 ng CA/10^7^ cells) or (**F**) equivalent amount of RT (5 ng of RT/10^7^ cells). Virus replication was monitored by using an ELISA to measure CA in the culture supernatant at 0, 3, 7 and 14 days post-infection. The mean values and standard deviations from an experiment performed in triplicate are shown. The experiment was repeated twice with similar results.

The RT W252 residue is largely invariant among published HIV-1 sequences but to our knowledge has not been investigated. Using an HIV-1_NL4.3_ proviral DNA plasmid, the RT W252A mutation was introduced in *pol* so that infectious virus could be produced. The HIV-1 wild-type and W252A RT mutant proviral plasmids were transfected into HEK293T cells, along with a control plasmid pCMV-Gluc2, and cell lysates and culture supernatants were collected at 48 h post-transfection. The expression of HIV-1 proteins in the cell lysate was examined by western blot using an anti-HIV-1 polyclonal antibody and virus particle production was determined by p24 ELISA from the culture supernatant. The levels of *Gaussia* luciferase were determined and used to normalize results for transfection efficiency. The levels, patterns and ratios of viral proteins in cell lysates were similar between the wild type and mutant (Fig 4A). Virus particle production of the W252A RT mutant in culture supernatant was slightly reduced compared to wild type virus (Fig 4B). Western blot of partially purified virions using an anti-HIV-1 polyclonal antibody or an anti-RT antibody showed similar protein staining patterns of viral proteins and RT (Fig 4C), suggesting that an W252A RT mutation did not significantly affect Gag-Pol steady-state levels or its cleavage by HIV-1 protease.

The thumb domain plays a role during reverse transcription to help properly position the viral nucleic acid through interactions that involve both the primer and template strands [23], so a W252A mutation could affect RT activity. To test this hypothesis, equal amounts of wild type or W252A RT mutant virus, normalized to CA levels, were used in a standard *in vitro* RT assay. Each virus sample was incubated in reaction mixtures containing primer, template and nucleotides for 2 h at 37°C. The results show that a W252A RT mutation reduced the level of RT activity by ~50% (Fig 4D). Hence a W252A RT mutation can down regulate both RT activity and the interaction between eEF1A and RT.

### The W252A RT mutation affects late steps of reverse transcription and virus replication

The effect of a W252A RT mutation on reverse transcription efficiency was assessed in infected cells. Jurkat cells were infected with HIV-1 normalized to one of two parameters: using an equivalent amount of CA or an equivalent amount of total RT activity. The cells were incubated with virus for 2 h at 4°C to enable virus attachment and then at 37°C for 4 h to initiate virus entry and reverse transcription. The cytoplasmic nucleic acids were isolated 4 h later and levels of viral early DNA and late DNA were measured by qPCR, and the values were normalized to the total mitochondrial DNA present in each sample. Heat inactivated viruses were used as a negative control. When the infections were normalized to CA, the W252A RT mutant virus produced early viral DNA at levels slight lower than wild type virus (p > 0.05; Table 3), but the levels of late viral DNA made by the W252A RT mutant virus was significantly reduced (p < 0.05). The results show that the efficiency of reverse transcription, defined as the ratio of sssDNA levels to second strand transfer DNA levels, was reduced by 2-fold in the mutant virus compared to wild type HIV-1 (Table 3). When the amount of virus used for infection was normalized to total RT activity, thereby increasing the input of W252A RT mutant virus relative to wild type, the level of early DNA made by the W252A RT virus was proportionally increased, but the relative efficiency of reverse transcription remained low compared to wild type HIV-1 (Table 3). As expected, replication of the W252A RT virus was much weaker than wild type in Jurkat cells (Fig 4E and 4F). To examine whether synthesis of late reverse transcription DNA was more sensitive to RT activity changes rather than early reverse transcription DNA levels, the efficiency of reverse transcription was assessed by wild type virus infection in the presence of an RT inhibitor, nevirapine. When nevirapine was used to inhibit RT activity by ~50%, synthesis of both early and late DNA products were equally affected so that the overall efficiency of reverse transcription was unchanged (Table 3). The combined results suggest that the W252A RT mutation affects late DNA synthesis due to changes in the RT-eEF1A interaction rather than due to reduced RT activity *per se*, and this significantly reduced HIV-1 replication.

**Table 3:** Analysis of reverse transcription by wild type or RT-W252A mutant virus in Jurkat cells.

		WT^1^ ^,^ ^2^ ^,^ ^3^ ^,^ ^4^	W252A^1^ ^,^ ^2^ ^,^ ^3^ ^,^ ^4^	p value^5^	WT + NVP^6^ ^,^ ^4^
Infection using equivalent amount of CA^8^	early DNA copy number	15,212 ± 3,226	11,350 ± 3,110	≥ 0.05	8,393 ±3,785
	late DNA copy number	3,244 ± 696	1,332 ± 216	< 0.05	1,912 ± 598
	late/early DNA %	21.3 ± 0.2	11.7 ± 0.6		22.7 ± .06
Infection using equivalent amount of RT^9^	early DNA copy number	9,780 ± 1,433	16,440 ± 3,745		NT^7^
	late DNA copy number	2,207 ± 336	2,152 ± 544		NT^7^
	late/early DNA %	22.6 ± 0.3	13.1 ± 0.4		NT^7^

^1^. The infection experiments were repeated at least three times and the mean copy numbers of DNA measured and standard deviations are shown;

^2^. HIV-1NL4.3;

^3^. HIV-1NL4.3-RT-W252A;

^4^. Heat inactivated virus samples measured less than 100 copies (subtracted);

^5^. The data sets analyzed were considered to be normal with an α value of > 0.05 standard deviations. P values from Welch’s two-tailed t test are shown, comparing experimental and control samples as indicated;

^6^. Nevirapine (NVP) was added at 40 nM which is the reported IC50 in cell culture;

^7^. NT = Not Tested;

^8^. CA measured by ELISA;

^9^. RT measured by *in vitro* RT assay.

### The W252A RT mutation significantly reduced the association of RT with eEF1A in infected cells

**Fig 5:**
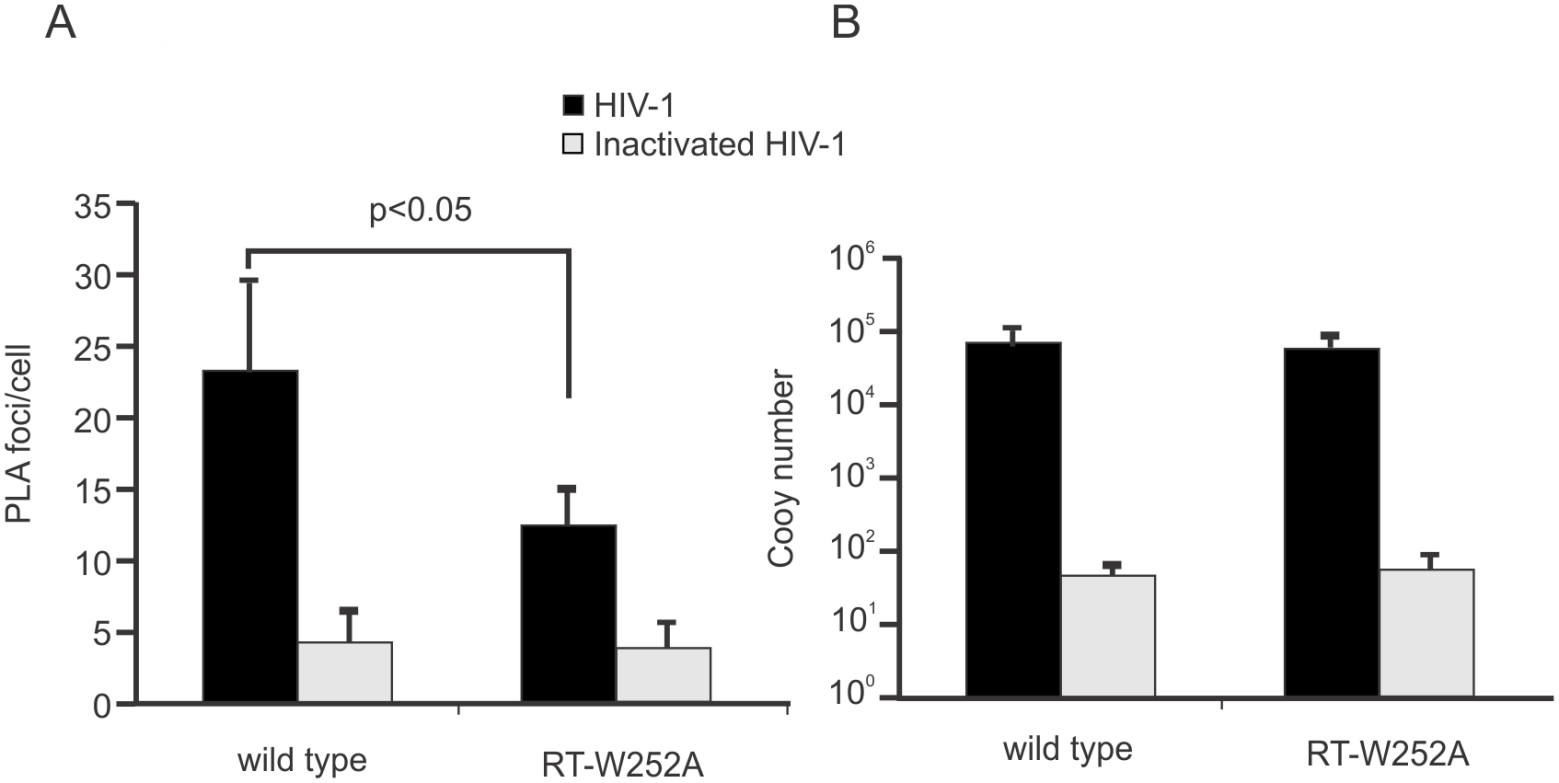
A W252A RT mutation reduces the association of RT with eEF1A in cells. Proximity ligation assays (PLA) were performed in TZM-bl cells infected with equivalent amounts of virus normalized to CA. Foci indicating association of RT with eEF1A were detected by fluorescence microscopy of cells infected with wild type or W252A RT mutant virus (bottom left panel), or with the same viruses that were heat-inactivated prior to infection. (**A**) Foci from PLA positive cells in 20 fields totaling at least 200 cells were quantified for each infection experiment. (**B**) Quantitative RT-PCR analysis of viral RNA in nevirapine-treated TZM-bl cells (to block reverse transcription of viral genomic RNA) infected with wild type or W252A RT HIV-1 or their heat-inactivated controls. The cytoplasmic RNA was extracted 2 h post-infection and measured by RT-PCR in triplicate. All values were normalized to cellular β-tubulin mRNA levels. All columns represent a mean value and standard deviation from at least three independent experiments. A p value for the wild type and W252A RT mutant HIV-1 outcome is shown.

Proximity ligation assays (PLA) were performed to examine the association of RT with eEF1A in infected cells. Previously we used PLA to show an association of HIV-1 RT with eEF1A in infected cells [9]. Similar experiments were performed here using equivalent amounts of wild type or W252A virus, normalized to CA, to infect TZM-bl cells. The cells were fixed at 4 h post-infection and mouse anti-RT and rabbit anti-eEF1A antibodies were used to target RT and eEF1A, respectively, which were detected using oligonucleotide-conjugated secondary antibodies. Heat-inactivated viruses that are defective for entry into cells were used to monitor non-specific foci, due to low levels of non-specific antibody staining. The number of foci was significantly reduced in cells infected by W252A RT virus compared with wild type virus (p <0.05; Fig 5A), indicating a reduced association of W252A RT with eEF1A. The degree of cell entry of each virus was similar, as indicated by similar levels of viral genomic RNA in cells post-infection (Fig 5B). The PLA data correlate to decreased association of W252A RT with eEF1A as observed in the co-IP assays (Fig 2G) and provide further evidence that a W252A mutation in RT impairs its interaction with eEF1A.

### The eEF1A inhibitor, didemnin B (Did B) inhibits HIV-1 reverse transcription

Did B is a marine natural product depsipeptide with antiviral and antitumor properties that can inhibit protein synthesis [24,25]. A proposed mechanism of action involves Did B irreversibly binding to a pocket in eEF1A domain 2, resulting in a conformational change that inhibits interaction of eEF1A with the nucleotide exchange factors eEF1B and eEF2 [25–27]. We hypothesized that binding of Did B to eEF1A may also inhibit reverse transcription via the RT-eEF1A interaction. To compare the effect of Did B, another protein synthesis inhibitor, cycloheximide (CHX), which blocks eEF2-mediated tRNA translocation by binding to the E site of the 60S ribosome [28], was used so possible effects on early HIV-1 replication could be discriminated from inhibition of translation.

**Fig 6:**
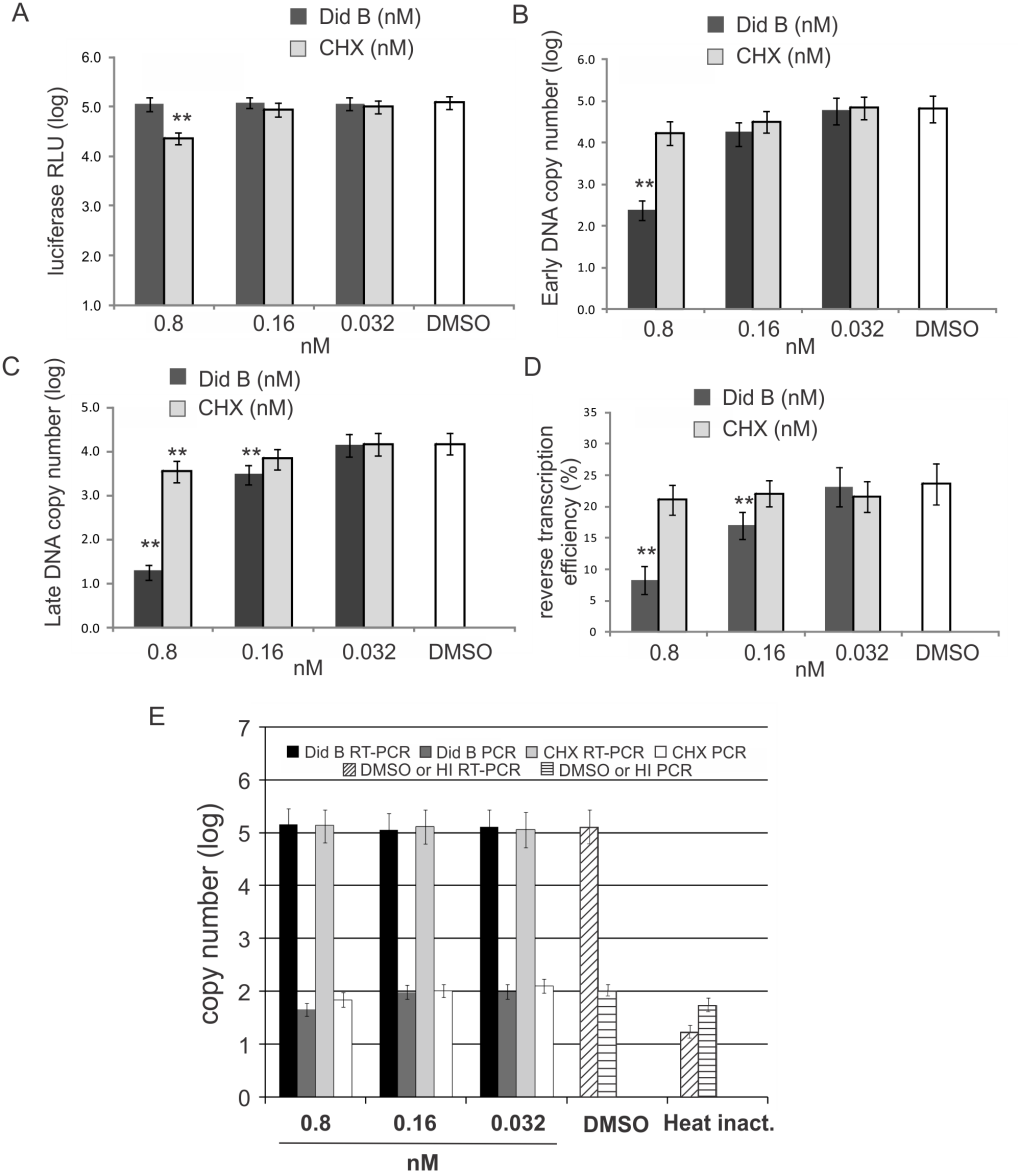
The eEF1A inhibitor didemnin B blocks HIV-1 reverse transcription. (**A**) Jurkat cells transfected with pCMV-Gluc 2 were treated post-transfection with media containing didemnin B (Did B) or cycloheximide (CHX) at concentrations as indicated. The levels of secreted *Gaussia* luciferase in the culture supernatants were measured 8 h post-treatment. (**B** and **C**) Jurkat cells were incubated with subnanomolar concentrations of Did B and CHX as indicated and then infected with HIV-1 at 4°C for 2 h and 37°C for 4 h. The total cytoplasmic DNA was collected and the levels of HIV-1 early (**B**) and late (**C**) reverse transcription products were measured by qPCR. (**D**) Reverse transcription efficiencies were calculated as the ratio of late to early viral DNA, expressed as a percentage. (**E**) The levels of HIV-1 RNA in cells treated with didemnin B (Did B) and cycloheximide (CHX). Jurkat cells were incubated with 100 nM nevirapine and concentrations of Did B and CHX as indicated at 37°C for 2 h followed by HIV-1 infection. The HIV-1 was incubated with cells at 4°C for 2 h and then at 37°C for 4 h. Heat inactivated virus was used as a negative control. The HIV-1 RNA was extracted from the cells and detected by RT-qPCR. A direct qPCR without RT from the RNA samples was performed to monitor for DNA contamination. All columns represent a mean value and standard deviation from at least three independent experiments. ** = *p* < 0.05.

Firstly, the effects of Did B and CHX on protein translation were measured in Jurkat cells. Jurkat cells were transfected with pCMV-Glu2 expressing secretable *Gaussia* luciferase and the culture medium was replaced 24 h post-transfection with media containing increasing subnanomolar concentrations of Did B and CHX (0.032 to 0.8 nM). At 8 h and 24 h post-treatment, Did B did not reduce levels of *Gaussia* luciferase in culture supernatant, while CHX modestly decreased levels when used at 0.8 nM (p < 0.05) compared to a DMSO control (Figs 6A and S8A), indicating that CHX had a greater negative effect on cellular translation. Cell proliferation was reduced using 0.8 nM of Did B, however a greater reduction of cell proliferation was detected at all concentrations of CHX used (S8B Fig). The data indicate that CHX is significantly more cytotoxic than Did B when used at 0.8 nM.

The effect of Did B and CHX on HIV-1 reverse transcription was then assessed. Jurkat cells were treated with equivalent concentrations of Did B or CHX for 2 h prior to infection. In the continued presence of the compounds, cells were infected with HIV-1 for 2 h at 4°C, then at 37°C for 4 h, after which the cytoplasmic nucleic acids were extracted and the levels of HIV-1 DNA in the samples were measured by qPCR. The results show that Did B strongly inhibited reverse transcription, especially at 0.8 nM, where DNA synthesis was reduced compared to mock (DMSO) treated cells (Fig 6B and 6C). CHX had a negligible effect on reverse transcription at the same concentration. Most noteworthy was a change in reverse transcription efficiency, as indicated by the amount of second strand DNA synthesis relative to the amount of sssDNA produced. The maximum reverse transcription efficiency was ~24% in DMSO treated cells, whereas reverse transcription efficiency in cells incubated in the presence of 0.8 nM Did B was reduced ~ 3-fold (Fig 6D). At a concentration of 0.16 nM, Did B had no detectable cytotoxicity (Figs 6A, S8A, and S8B), but still reduced reverse transcription efficiency (Fig 6D). No significant change in reverse transcription efficiency was observed in cells treated with CHX compared to the DMSO treated control. The levels of mitochondrial DNA, as measured by PCR of cytochrome c oxidase subunit II (COXII), were similar in all samples (S9A Fig), indicating that equivalent amounts of samples were assayed. Furthermore, we excluded any inhibition of HIV-1 entry by both Did B and CHX treatments (Fig 6E) and any direct effects of Did B and CHX on RT activity (S9B Fig). The results demonstrate Did B can inhibit early and late HIV reverse transcription, but that the effect of Did B on late DNA synthesis is greater, resulting in the reduction of reverse transcription efficiency. This outcome is consistent with a role we previously described for eEF1A in supporting the stability of the reverse transcription complex and completion of the reverse transcription process [9].

### Did B induces instability of the wild type but not W252A mutant reverse transcription complex

**Fig 7:**
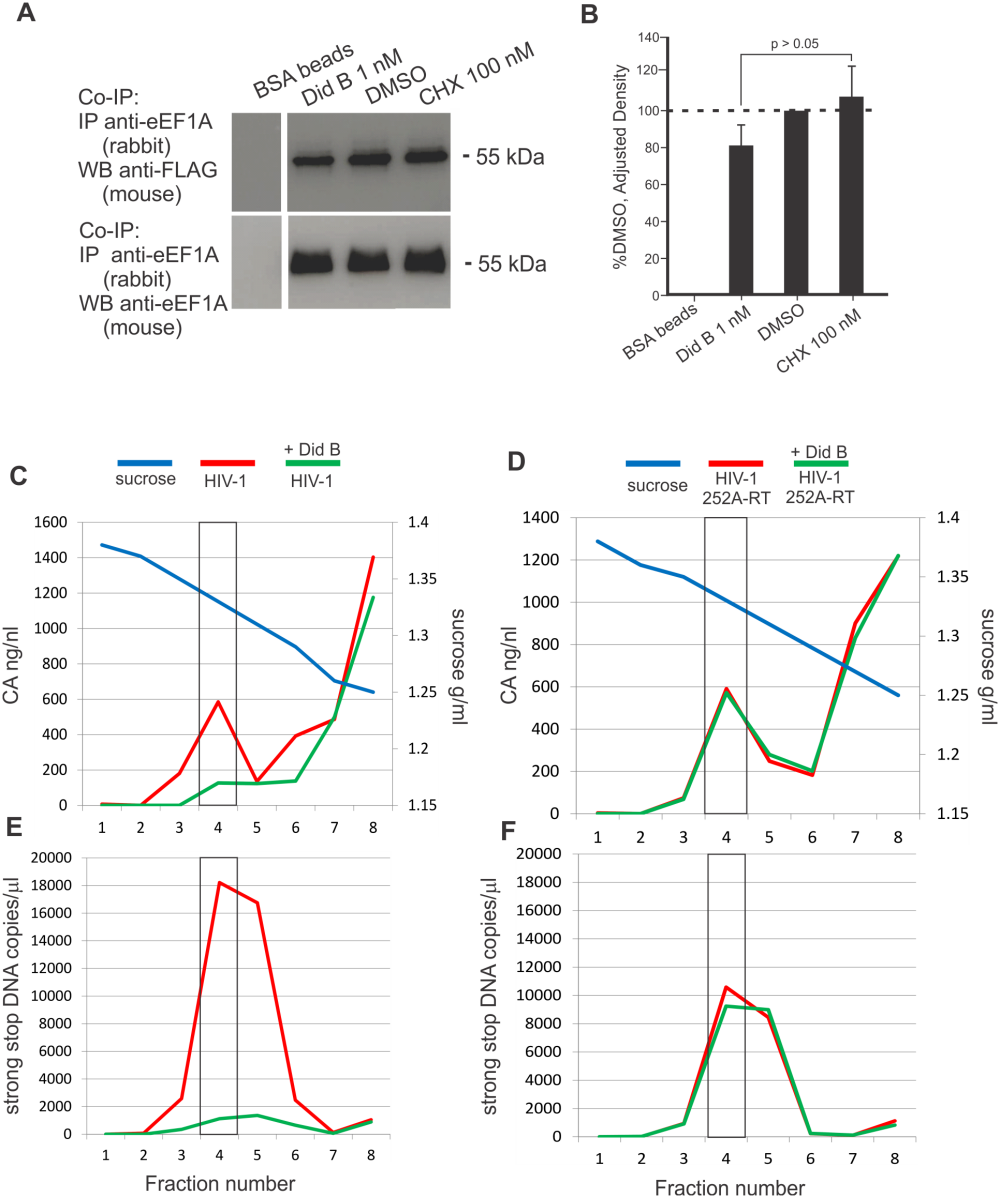
The effect of didemnin B on RT-eEF1A interaction *in vitro* and viral RTCs in cells. (**A**) Cell lysates from HEK293T cells transiently expressing HIV RTp51-FLAG were incubated for 30 min at 37°C with concentrations of Did B, CHX or DMSO as indicated. Immunoprecipitation was performed using a rabbit anti-eEF1A antibody and visualized by western blot using anti-FLAG (upper panel) or mouse anti-eEF1A antibodies (lower panel). The experiment was performed three times with similar results. (**B**) Western blots in (**A**) were analyzed by densitometry. Normalized density values for RTp51 were calculated by dividing the relative density of RTp51 in each sample lane by the relative density of the eEF1A in the same lane [45]. The columns represent the mean normalized densities and standard deviations of three independent experiments relative to the DMSO treated value. Student’s two-tailed T test was used to calculate statistical significance (**C**—**F**) HEK293T cells were treated with Did B at 0.8 nM or with DMSO carrier. The cells were infected with VSV-G psuedotyped wild type (**C** and **E**) or W252A RT mutant (**D** and **F**) HIV-1. A lysate was prepared and viral RTCs were purified by isopycnography using a 20% to 70% linear sucrose gradient. Fractions collected from the tube bottom were assayed for (**C** and **D,** left y-axis) CA by ELISA and (**E** and **F,** left y-axis) viral strong stop DNA by qPCR. The density of the sucrose in each fraction (**C**–**F**, right y-axis) is indicated by the blue line. The box in each graph indicates a fraction with a density of 1.33 g/ml. The experiment was repeated twice with similar results and a representative outcome is shown.

Did B is believed to induce conformational changes in eEF1A [26,27], and this may affect interaction with RT and functionality of the RT-eEF1A complex. To determine if Did B could directly affect the eEF1A interaction with RT, cell lysate from cells expressing HIV-1 RTp51-FLAG were incubated with 1 nM Did B, or 100 nM CHX or DMSO as controls. eEF1A was immunoprecipitated from these treated lysates using an anti-eEF1A antibody and the captured proteins were analyzed by western blot using an anti-FLAG antibody. The results show that treatment with 1 nM Did B resulted in a small decrease in co-IP of RT (Fig 7A), but the difference was not statistically significant (Fig 7B).

To test the effect of Did B on RTCs and reverse transcription, large scale infections of HEK293T cells were performed using VSV-G pseudotyped wild type and W252A mutant HIV-1. HEK293T cells were incubated with 0.8 nM Did B or DMSO carrier for 2 h. VSV-G pseudotyped HIV-1 was incubated with cells at 4°C for 2 h to allow virus attachment to cells, then at 37°C for 4 h. Cell lysates were made from the infected cells and fractionated by isopycnography using a 20% to 70% linear sucrose gradient. The analysis shows the bottom eight fractions that include a sucrose density of 1.33 g/ml, which corresponded to fraction 4 (F4), that contains viral RTCs [29] (Fig 7C–7F). Viral CA and DNA were observed with a peak in F4 in agreement with previous studies [9] and indicative of RTCs (Fig 7C–7F, boxed). While peak profiles for the wild type and mutant HIV-1 were similar (Fig 7C vs. 7D, and 7E vs. 7F), the W252A mutant HIV-1 made less early DNA than wild type HIV-1, in agreement with previous experiments. Did B reduced CA levels of wild type HIV-1 by 5-fold (Fig 7C, red vs. green lines), and early DNA levels by 13-fold (Fig 7E, red vs. green lines). Did B had a greater effect on early DNA synthesis in our previous experiments (Fig 6B). However, Did B had no dramatic effect on the W252A mutant HIV-1 CA or viral DNA levels in any fraction (Fig 7D and 7F, red vs. green lines). We also measured the levels of late DNA in F4 by qPCR. The results averaged from two experiments show that the efficiency of wild type HIV-1 reverse transcription was ~21% in untreated cells and ~11% in Did B treated cells. The W252A mutant reverse transcription efficiency was ~12–13% in either the presence or absence of 0.8 nM Did B (Table 4). Based on these observations, we propose that Did B inhibits reverse transcription by binding to eEF1A thereby affecting its interaction with the RTC.

**Table 4:** Analysis of lysates of from mock and Did B-treated cells for viral DNA in RTC fraction 4.

	WT^1^ ^,^ ^3^ +DMSO	WT^1^ + Did B	p value^4^	W252A^2^ ^,^ ^3^ + DMSO	W252A^2^ + Did B	p value^4^
early DNA copy number/μl	13304 ± 4933	1124 ± 457	<0.05	6788 ± 3780	7090 ± 4462	≥ 0.05
late DNA copy number/ μl	2781 ± 856	122 ± 48	<0.05	829 ± 368	872 ± 522	≥ 0.05
late/early DNA%	21.2 ± 2.1	10.9 ± 1.02	< 0.05	12.7 ± 2.2	12.5 ± 0.6	≥ 0.05

^1^. VSV-G pseudotyped HIV-1NL4.3;

^2^. VSV-G pseudotyped HIV-1NL4. 3-RT-W252A;

^3^. Heat inactivated virus samples measured no detectable copies. The experiment was repeated twice with similar outcomes. Mean values and standard deviations of combined experiments are shown;

^4^. P values from Welch’s two-tailed t test are shown, comparing experimental (Did B) and control samples (DMSO) as indicated.

## Discussion

Previously we demonstrated that the eEF1 complex associates with and had functional importance for the HIV-1 RTC [9]. Here we provide evidence that eEF1A binds tightly and specifically to RT and directly regulates reverse transcription. We previously showed that two subunits of the eEF1 complex, eEF1A and eEF1G, could interact with the RTC components RT and IN and affect RTC stability and its ability to complete late steps of reverse transcription. Both HIV-1 RT and IN can be co-immunoprecipitated by antibodies specific for eEF1A or eEF1G [9]. However, co-IP experiments cannot discriminate between direct or indirect protein interactions. Here, BLI assays were used to investigate direct binding between RT or IN with individual components of the eEF1 complex including eEF1A, eEF1G, eEF1D and eEF1B. BLI assays detected strong binding between eEF1A and RT, whereas weak or no binding was observed between other eEF1 subunits and RT or IN. For unclear reasons, downregulating eEF1G using siRNA usually resulted in reduced level of eEF1A, which may partially explain why down regulating eEF1G also reduces reverse transcription [9]. Interestingly, eEF1A levels are downregulated by siRNA knockdown of eEF1Bα in cells [30]. A remarkably strong K_D_ of 3–4 nM between the RT heterodimer and eEF1A was measured under the conditions tested, which is a stronger interaction constant than that described for eEF1A-Gag [12]. The results indicate that interaction between eEF1A and RT actuates the previously described association of the eEF1 complex with the RTC [9].

BLI has the ability to detect specific binding of proteins between a probe and an analyte in complex mixtures of molecules such as a cell lysate. Given the relative abundance of eEF1A in cells and its strong affinity for RT, it is not surprising that knockdown of eEF1A by siRNA in cell lysates correlated with decreased levels of RT binding in BLI assays, which could be compensated by adding purified eEF1A to an eEF1A-depleted cell lysate. Given the intense interest in cellular proteins that interact with viral proteins, reports of other cellular proteins associating with RT are surprisingly rare [31–34]. To our knowledge, only a few other cellular RT binding proteins, including AKAP149 and APOBEC3G, are reported to enhance or inhibit reverse transcription, respectively, through direct interactions with RT [35,36]. The data presented here provide evidence that eEF1A is an important cellular RT binding protein required for early HIV-1 replication.

Our experiments show that the RT thumb and connection domains are required for interaction with eEF1A. We observed reduced protein interactions in co-IP experiments when regions within the thumb domain or the entire connection domain were deleted. Given the strong binding between RT and eEF1A, multiple contacts between the two proteins are likely. The W252A and L303A mutants, in particular, are interesting because their side-chains are buried in the RT core and unlikely contact points between RT and eEF1A, and their individual mutation to alanine imparts a localized structural change that is sufficient to disrupt RT interaction with eEF1A. An HIV sequence database search indicates that W252 and L303 residues are largely invariant residues demonstrating their importance in RT structure and function, and we were unable to find other studies investigating RT with mutations in these positions. Our analysis of the W252A RT mutant provides a proof of concept that negatively impacting the RT-eEF1A interaction leads to reduced reverse transcription efficiency and HIV-1 replication. Our modeling and MAPPIT experiments testing dimerization of RT subunits support the interpretation that W252A acts by inducing a localized change in RT structure that does not affect RT dimerization. The modeling also predicts that a T253A mutation will not affect RT structure and RT protein containing this mutation efficiently binds to eEF1A in co-IP assays indicating specificity for the W252 region. Further analysis of the thumb and connection domains and their effect on RT interaction with eEF1A will be fertile ground for future research.

**Fig 8:**
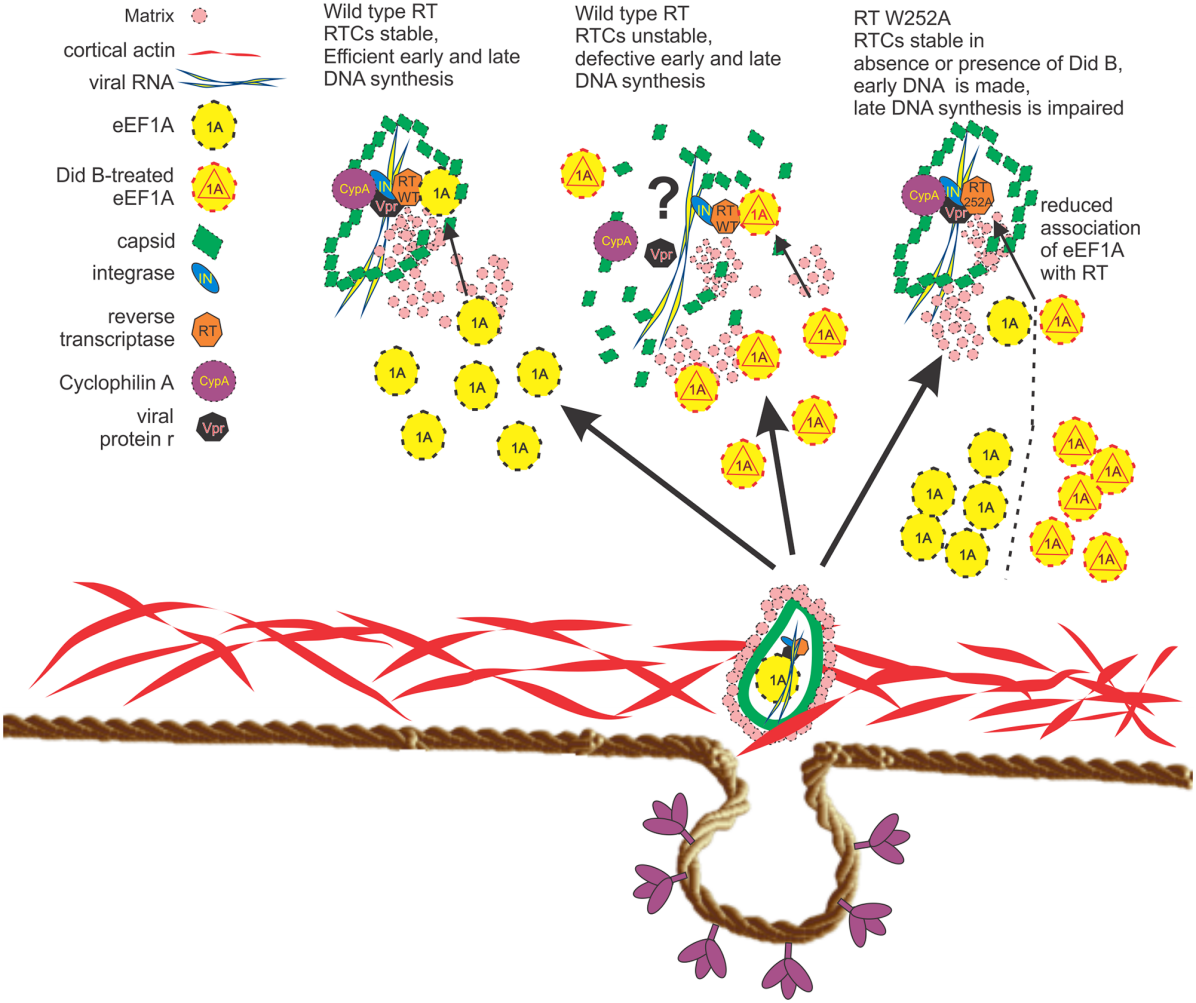
Did B inhibits early HIV-1 replication by targeting the RT-eEF1A interaction. Immediately following fusion of the viral and cellular membrane, the viral core and matrix are released into the cytoplasm. Subsequently the viral core undergoes uncoating and reverse transcription. Here we propose that eEF1A is recruited to the RTC by a direct interaction with RT that increases the efficiency of reverse transcription completion. It is unclear if virion eEF1A [12], target cell eEF1A or both are required. HIV-1 containing a W252A RT mutant has decreased ability to interact with eEF1A. Treating cells with Did B generates pools of eEF1A-Did B as well was RT-eEF1A-DidB complexes. The RT-eEF1A-Did B complex does not support uncoating and reverse transcription and results in decreased RTC levels and reduced DNA synthesis. The status of other RTC protein interactions requires experimental confirmation (as denoted by a “?”). However, a W252A RT mutant HIV-1 is unaffected by Did B because its interaction with eEF1A, or with a eEF1A - Did B complex, is downregulated and catalyzes reverse transcription in either the presence or absence of Did B albeit at reduced efficiency.

Didemnin B is a depsipeptide isolated from a Caribbean tunicate that has reported antiviral, anti-tumor, and anti-translation effects [24–26]. When comparing matched subnanomolar concentrations of CHX and Did B, we found that CHX is a stronger translation inhibitor with greater cytotoxic effects in Jurkat cells compared to Did B. However, only Did B reduced the efficiency of reverse transcription, the ratio of the late to early viral DNA reverse transcription products. Interestingly Did B strongly decreased RTC levels and reduced DNA synthesis in cells infected with wild type HIV-1 but not with the RT W252A mutant. It suggests that eEF1A plays a broader role in reverse transcription, a concept supported by the observation that eEF1A is specifically packaged in HIV-1 virions [12], and can interact with the HIV-1 5’ UTR genomic RNA as well as with RT [37]. Our experiment supports a model (Fig 8) in which Did B irreversibly binds to eEF1A [25], associated with RT leading to instability of the RTC and viral core supporting the RTC, thereby negatively effecting both early and late DNA synthesis during reverse transcription. However Did B has a greater effect on late DNA synthesis compared to early consistent with the observation that the RT–eEF1A complex is particularly important for the late phase of reverse transcription, in agreement with our previous study [9]. However RTCs with a RT W252A mutant, which can interact with eEF1A but at reduced levels compared to wild type RT (Figs 2 and 3), maintain an RTC complex capable of strong stop DNA synthesis whereas late DNA synthesis occurs at reduced efficiency (Table 4). The contrasting Did B-sensitivity of HIV-1 wild type and W252A mutant RT indicates that Did B affects reverse transcription through the RT-eEF1A interaction and not by off-target effects.

Studies of tombusvirus, a non-enveloped positive sense single-stranded RNA plant virus, provide evidence that Did B inhibits viral replicase activity by blocking replicase formation through binding to eEF1A [38]. The fact that eEF1A can assume a partially unstructured conformation [39] may underlie its ability to interact with many different viral proteins and RNAs. While Did B strongly impaired reverse transcription, it is not a therapeutic drug due to toxicity. However, eEF1A is targeted by other compounds without toxic effects [40,41]. Hence, detailed knowledge of the RT-eEF1A interaction could lead to the identification of agents that impede their interaction without detrimental effects to the cell, thereby nominating well tolerated, lead anti-viral candidates that target a druggable host-pathogen interaction.

## Materials and Methods

### Cell lines and virus culture

HEK293T (ATCC) and TZM-bl (NIH AIDS Reagent Program) cell lines were grown in Dulbecco's modified Eagle's medium supplemented with 10% heat-inactivated fetal bovine serum, penicillin (50 I.U./ml)-streptomycin (50 μg). Jurkat (Clone E6-1, ATCC) cells were grown in RPMI 1640 supplemented with 10% fetal bovine serum, penicillin (50 I.U./ml) and streptomycin (50 μg/ml). All cell lines were incubated at 37°C in 5% CO_2_. A stock of HIV-1_NL4.3_ and the same strain with a mutated RT gene, HIV-1 _NL4.3_-pol-W252A, were generated by transfection into HEK293T cells of the corresponding proviral DNA using Lipofectamine 2000 (Invitrogen, Carlsbad, CA) according to the manufacturer's recommendations. Cell culture supernatants were collected at 48 h post-transfection and centrifuged (200 × *g*, 10 min), and the supernatant was filtered (0.45 μm) and stored in 1 ml aliquots at −80°C.

### BLI assay

Full-length human proteins expressed in HEK293 cells including eEF1A1, 1B2, 1G and 1D proteins having C-terminal MYC/DDK tags were purchased from OriGene (OriGene Technologies, MD, USA) and recombinant 6×His-tagged HIV-1 RT p51, RT p66, IN and Gag produced in *E*. *coli* were gifts from Stuart LeGrice, Duane Grandgenett and Johnson Mak, respectively. The proteins were biotinylated using EZ-Link Sulfo-NHS-Biotin following the manufacturer’s instructions (Pierce Biotechnology, IL, USA). The biotinylated proteins were immobilized onto streptavidin-coated biosensors (Pall ForteBio, CA, USA). A standard kinetic buffer (1 mM phosphate, 15 mM NaCl, 0.002% Tween-20 and 0.1 mg/ml gelatine) was used in all experiments unless noted. The association (ka) of the two proteins was measured by incubating ligand biosensors (biotinylated protein) into kinetic buffer containing protein (analyte) with 1000 rpm shaking in the OctetRed system. Concentrations of analyte ranging from 90 nM to 10 nM were assayed. The dissociation (kd) was determined by moving the ligand biosensor from kinetic buffer containing analyte to the standard kinetic buffer.

When cell lysates were used in BLI assay, the total protein concentration was adjusted to 0.5mg/ml using kinetic buffer and the biosensors immobilized with RT51/66. In add back experiment, the purified eEF1A protein was added into siRNA treated cell lysate to the final concentration of 45 nM, or the same amount of BSA was added other cell lysates.

### siRNA treatment

siRNA (Sigma-Aldrich) targeting eEF1A (siRNA ID: SASI_Hs02_00331772 and 00331773) and control siRNA (SIC001) were applied to HEK293T cells by large-scale reverse transfection using Lipofectamine RNAiMAX according to the manufacturer’s instructions (Invitrogen). The cell lysates were prepared using a Dounce homogenizer in S100 buffer at 48 h of post-transfection and cleared by centrifugation 16,000g for 30 min at 4°C as previously described [9].

### Plasmids

For expression of HIV-1 RT p51, p66, IN and Gag separately in cells, the coding DNA sequences were amplified from pHGPsyn construct [42], which contains a codon optimized Gag-Pol sequence that was inserted into pDONR vector using Gateway system (Invitrogen, USA) by a BP recombination reaction. The inserts were then transferred into destination mammalian expression vectors that contain FLAG or V5 and 6×His tags by LR recombination reactions. Internal deletions and mutations of RT in the destination vector were made by inverse PCR. Inverse PCR is also used to make RT mutated proviral plasmid using pGCHIV-1-NL4-3 construct as a template [43]. The secreted *Gaussia* luciferase expression construct, pCMV-Gluc 2 was purchased from NEB (NEB, USA). The sequences of the primers used are available on request.

### Virus infection, DNA extraction and analysis of reverse transcription DNA synthesis

HIV-1_NL4.3_ virus stock was treated with DNase I at 37°C for 30 min to remove any DNA contamination prior to adding to cells. Jurkat cells were incubated with HIV-1_NL4.3_ virus in presence of 8 μg/ml polybrene at 4°C for 2 h and then 37°C for 4 h. The cells were then washed three times with 0.1 mM EDTA PBS, and lysed using Glo Lysis Buffer (Promega). The lysate was centrifuged (20,000 x *g*, 10 min). The supernatant was extracted once with an equal volume of phenol:chloroform:isoamyl alcohol (25:24:1) and once with chloroform. The extracts were ethanol precipitated, washed with 70% (vol/vol) ethanol, and resuspended in 0.1 mM EDTA. The early and late reverse transcription products were analyzed by quantitative PCR as elsewhere described [6].

### Co-immunoprecipitation (Co-IP) and protein analysis

Co-IP was performed as elsewhere described [9]. Briefly, cells expressing various wild type or mutant HIV-1 proteins having V5 or FLAG epitope tags were lysed in S100 buffer (10 mM Tris, pH 7.4, 1.5 mM MgCl_2_, 10 mM KCl, 1× complete protease inhibitor cocktail [Roche], and 0.5 mM β-mercaptoethanol) using a Dounce homogenizer. The lysate was cleared by centrifugation in a microfuge (4°C, 20,000 x *g*, 10 min). The supernatant was incubated with anti-eEF1A rabbit antibody or rabbit IgG coupled Dynabeads Protein G (Invitrogen, USA) for 2 h at 4°C. The immunoprecipitate was washed three times with S100 buffer plus 0.1% Triton-x100 followed by western blot analysis using anti-FLAG, anti-V5 antibodies or a mouse anti-eEF1A antibody (Santa Cruz) as indicated. Where indicated western blots were probed with anti-HIV-1 immunoglobulin (HIV-IG, obtained through the NIH AIDS Reagent Program, Division of AIDS, NIAID, NIH: Catalog #3957, HIV-IG from NABI and NHLBI) and HIV-1 RT antibody (39/4.12.2) (Santa Cruz). The Agilent 2100 system and Protein 230 kits were used to evaluate protein lysate used in the study according to the manufacturer’s instructions. Some western blots were analyzed using ImageJ software [44] following published procedures [45].

### Purification of recombinant RT wild type and mutants

The plasmids containing wild type or mutant RT p66 in pDEST 40 vector (Invitrogen, USA) were transformed into BL21 AI bacterial cells (Invitrogen, USA). The cells were cultured to the density of OD600 ~0.4 and the protein expression was induced by the final concentration of 1mM IPTG and 0.2% L-arabinose (Sigma, USA). The protein purification was carried out using the HisLink protein purification system according the product protocol (Promega, USA). The purified proteins were dialyzed using MINI dialysis unit (Thermo Scientific, USA) in PBS and examined by SDS PAGE and Coomassie blue staining.

### Proximity ligation assay (PLA)

PLA assay was performed as elsewhere described [9]. Briefly, TZM-bl cells were incubated with HIV-1_NL4.3_ or HIV-1_NL4.3_-pol-W252A (equivalent to 500 ng CA) at 4°C for 2 h to allow virus attachment. Heat-inactivated virus (85°C, 15 min) were used as a negative control. The cells were then incubated at 37°C for 4 h to permit virus fusion with the cells and reverse transcription. Cells were fixed using 4% paraformaldehye followed by permeabilization using acetone. Duolink proximity ligation assays were performed using a mouse monoclonal antibody to detect HIV-1 RT in conjunction with a rabbit antibody to eEF1A (Santa Cruz). Otherwise PLA reactions were performed according to the manufacturer’s instructions (Sigma-Aldrich). DAPI stain was used to visualize the nuclei. Cells were visualized using a DeltaVision Core imaging system. Maximum-intensity projections of deconvolved images were analyzed using Duolink Image Tool software. More than 200 cells from at least 20 fields were analyzed for each experiment.

### Structure-based analysis of the HIV-RT p66/p51 mutations

A BLAST search [46] was undertaken for Gag-Pol reverse transcriptase sequence 588–1147 (i.e. the p66 sequence, incorporating p51) against the Protein Data Bank (PDID B) [47] using an E-value cut-off 10^−20^. A total of 406 equivalent or related structures/domains were retrieved and the X-ray structure with the highest resolution among them (PDID B ID: 4G1Q) [15] that matched the lengths of the p66/p51 sequence was used for further analysis.

The empirical forcefield FoldX [16,48] under the YASARA [49,50] molecular visualization program was used to estimate the free energy difference (stability change) upon mutagenesis from wild-type. Mutagenesis of W252A and T253A was undertaken *in silico* with the individual p51 and p66 subunits, and the p51/p66 heterodimer, independently. The ‘*RepairPDB’* option followed by the ‘*Mutate residue*’ option of FoldX was used to calculate the stability change with default parameters (Number of runs: 3; pH: 7; Temperature: 298 K; Ionic strength: 0.05 M; VdW design: 2). All resulting energies are expressed in kcal/mol. The result is the delta Gibbs free energy (ΔΔG) of mutation expressed in kcal/mol; average of 3 calculation runs is reported. The error margin of FoldX is ~0.5 kcal/mol, so changes in that range are considered insignificant. The prediction decision on whether the mutation stabilises or destabilises the structure is based upon [16,48] where; no effect on structural stability: ΔΔG is < 1.6 kcal/mol, and severely reduced structural stability: ΔΔG is > 1.6 kcal/mol.

Inter and intra-protein residue interactions were determined using the Protein Interactions Calculator (PIC) [51]. ASAView [52] was used to determine residue solvent accessibility. PyMol (http://www.pymol.org) was used for visualization and analysis.

### HIV-1 CA ELISA and *in vitro* RT assay

HIV-1 CA in the culture supernatant was measured by using a RETROtek HIV-1 CAp24 antigen ELISA (Zeptometrix, USA), according to the manufacturer's instructions. For RT analysis, after the addition of detergent to virions, an RT assay (Roche) was used to measure RT activity released from virions using a poly(A):oligo(dT)_15_ template following the manufacturer's instructions.

### Translation inhibitor experiments

Did B was kindly provided by the Natural Products Branch, NCI (NSC 325319, Bethesda, MD, USA), and CHX was purchased from Sigma Aldrich. Both chemicals were dissolved in DMSO for stock. To monitor translation of proteins, Jurkat cells were transfected with pCMV-Gluc 2 plasmid and seeded in 24-well plate. At 5 h post-transfection, serially diluted stocks of Did B and CHX were added at concentrations of 0.032, 0.16 and 0.8 nM. The luciferase activity in culture supernatant was measured after 8 h and 24 h of treatment using Biolux Gaussia luciferase Flex Assay kit (NEB). Cell proliferation was monitored using CellTiter 96 AQueous One Solution Cell Proliferation assay (Promega), as instructed by the manufacturer.

### HIV-1 entry assay

TZM-b1 or Jurkat cells were treated with nevirapine at 1 μM/ml for 2 h incubated at 37°C and with HIV-1 (wild type or mutant) for 2 h at 4°C to facilitate virus attachment and then at 37°C for 2 h to initiate fusion and entry. Cellular nucleic acids were collected using Trizol reagent (Life Technologies) and the RNA fraction was column purified using a Direct-zol mini-prep kit (Zymo Research) and treated on the column with DNase I prior to elution. The purified RNAs were used in RT-PCR reactions both with and without SuperScript III reverse transcriptase (Life Technologies) using random hexamer oligonucleotides for the first strand DNA synthesis. Second strand DNA and PCR was performed using oligonucleotides specific for full length viral mRNA as previously described [4]. A no template control was included in each experiment.

### Mammalian protein-protein interaction trap (MAPPIT)

The interaction of HIV RT p51 and p66 subunits was performed using MAPPIT system as previously described with modification [19,20]. Briefly, RTp51 and its mutants were inserted into the bait vector using BamH I and Not I cut site. RTp66 and its mutant were inserted into the prey vector using EcoR I and Xho I cut site. 293T cells were grown in 12 well-plate and transfected using Xtremegene (Roche) with 500 ng bait plasmid (either wild type or mutant HIV-1 RTp51), 500 ng prey plasmid (either wild type or mutant HIV-1 p66), 100 ng of reporter plasmid (pXP2d2-rPAP1-luciferase) and 100 ng of a plasmid (pCMV-GLuc2) to monitor transfection efficiency. A final concentration 100ng/ml of leptin (recombinant mouse leptin, R&D systems, USA) was added to the cells at 24 h post-transfection. The culture supernatant and cells were collected after additional 24 h. Gaussia luciferase was measured from culture supernatant to normalize transfection efficiencies and the firefly luciferase was measured from cell lysate to determine the RTp51 and RTp66 interaction.

### RTC purification and sucrose gradient

Purification of HIV-1 RTCs was carried out as previously described [9], with minor modifications. HEK293T were treated with 0.8 nM Did B or same volume of DMSO for 2 h at 37°C and then incubated with same amount (equivalent to Cap24), DNAseI treated VSV-G pseudotyped HIV-1_NL4.3_ or HIV-1 _NL4.3_-pol-W252A stock for 2 h at 4°C with polybrene (80 μg/mL) and then incubated at 37°C for 4 h. Heat-inactivated virus were used as a negative control. The RTCs were prepared as previous described [53] and placed on top of a linear sucrose gradient (20–70%, wt/wt) and subjected to centrifugation at 145,000 × g for 18 h at 4°C. Eight fractions were collected from the tube bottom and assayed by qPCR for HIV-1 negative strand strong stop DNA as previously described [9] and by ELISA for HIV-1 CA (Zeptometrix Corporation).

### Statistical analysis

Statistical analyses were performed using the Student’s *t*-test from at least three independent experiments or measurements. Statistical significance was set at p < 0.05.

## Supporting Information

S1 Fig Sensorgram of association and dissociation of eEF1A1, eEF1B, eEF1D and eEF1G with HIV RT. Biotinylated (A) eEF1A1, (B) eEF1B, (C) eEF1D and (D) eEF1G were immobilized on biosensors. The association and dissociation with various concentrations of HIV RTp66/p51 were measured respectively on the OctetRed system. Data are representative of three independent experiments.
(PPTX)

S2 Fig Sensorgram of association and dissociation of eEF1A1 with subunits of RT, the RT heterodimer and Gag. Biotinylated eEF1A1 was immobilized on biosensors. The association and dissociation of eEF1A with various concentrations of HIV RT subunit (A) p51, (B) p66, (C) p66/p51 heterodimer and (D) Gag were measured with the OctetRed system. Data are representative of three independent experiments.
(PPTX)

S3 Fig eEF1A can co-immunoprecipitate with RT. FLAG-tagged RT51 was expressed in HEK293T cells by plasmid transfection. Cell lysates were prepared 24 h post-transfection and subjected to immunoprecipitation using an anti-FLAG antibody. The detection of eEF1A (A), eIF3A (B) and RT (C) in cell lysate (left column) and the co-immunoprecipitated eEF1A (right column, top panel) were detected by western blot. A lysate from mock-transfected cells was used as a control.
(PPTX)

S4 Fig Ribbon diagrams representing the highest resolved crystal structure of the p66/p51 heterodimer (PDB B ID: 4G1Q). The W252 is located in the loop region just before the first alpha-helix of the thumb domain and is shown in sphere representation (red) within each subunit.
(PPTX)

S5 Fig Association of eEF1A with purified RT66 and mutants. Purified wild type RT p66 and mutant RT W252A, L303A mutants were analyzed using SDS-PAGE followed by Coomassie blue staining (A). In BLI assay, purified eEF1A was immobilized on the biosensors and incubated in solutions containing purified RT. The association maximums of 90 nM of wild type RT and mutants was compared (B). The data is presented as a mean value ± standard deviation from 3 independent experiments. *p<0.05.
(PPTX)

S6 Fig Mammalian protein-protein interaction trap (MAPPIT) is a cytokine receptor-based mammalian two hybrid method for analysis of protein-protein interactions. A leptin receptor deficient for STAT3 recruitment (red) is C-terminally to a bait protein (orange), in this case either RTp66 or RTp51. A flexible spacer (green) separates the fused proteins. A prey protein (purple) is N-terminally fused to a gp130 chain with four functional STAT3 recruitment sites. In the presence of ligand, interaction between the bait and prey leads to complementation of STAT3 signaling and activation of a reporter luciferase gene expressed by the rPAP1 promoter.
(PPTX)

S7 Fig The RT mutations W252A and T253A do not affect RT dimerization. The dimerization of RTp66 and RTp51 was investigated by MAPPIT. The pXP2d2-rPAP1-luciferase reporter plasmid was co-transfected with combinations of wild type or mutant, W252A (252A) and T253A (253A), RTp51 bait and RTp66 prey expression plasmids into HEK293T cells as shown. A bait construct having the myeloid differentiation primary response protein 88 (hMvD88) and a prey construct having SV40 large T antigen (SVT) were used as negative controls. Leptin (100 nM) was added to the cells at 20 h post-transfection and the cellular lysate was prepared 24 h later and used in luciferase assays. The data is presented as a mean value ± standard deviation from at least 3 independent experiments.
(PPTX)

S8 Fig Translation inhibition and cytotoxicity of didemnin B (Did B) and cycloheximide (CHX) in Jurkat cells. (A) The plasmid pCMV-Gluc2 was transfected into Jurkat cells for luciferase expression and Did B or CHX were added at concentrations as indicated. The levels of luciferase in culture supernatant were measured 24 h after treatment. (B) Jurkat cells were incubated with concentrations of Did B and CHX as indicated for 24 h at 37°C and then incubated with CellTiter 96 AQueous One Solution Cell Proliferation solution for 2 h at 37°C. The absorbance was measured at 490nm in a 96-well plate reader. The data is presented as a mean value ± standard deviation from at least 3 independent experiments. *p < 0.05
(PPTX)

S9 Fig Effect of Did B and CHX on cytoplasmic nucleic acid sample COXII levels and in vitro RT activity. (A) The copy number of COXII in DNA samples extracted from Did B and CHX treated cells. Jurkat cells were incubated with various concentrations of Did B and CHX followed by HIV-1 infection. The cytoplasmic nucleic acids were extracted from cells and COXII levels were measured by qPCR. (B) *In vitro* RT activity in the presence of Did B, CHX and nevirapine (NVP). RT (0.5 ng) was incubated with concentrations of Did B, CHX and NVP using the Roche Reverse Transcriptase Assay (colorimetric). The data is presented as a mean value ± standard deviation from at least 3 independent experiments.
(PPTX)
