# Supplementary material for: Specific Interaction between eEF1A and HIV RT Is Critical for HIV-1 Reverse Transcription and a Potential Anti-HIV Target
Source: PLoS Pathog. 2015 Dec 1;11(12):e1005289. doi: 10.1371/journal.ppat.1005289 (PMC4666417; doi:10.1371/journal.ppat.1005289)
Supplement: S2 Fig — Biotinylated eEF1A1 was immobilized on biosensors. The association and dissociation of eEF1A with various concentrations of HIV RT subunit (A) p51, (B) p66, (C) p66/p51 heterodimer and (D) Gag were measured with the OctetRed system. Data are representative of three independent experiments. (PPTX) [file ppat.1005289.s002.pptx]

## Slide 1
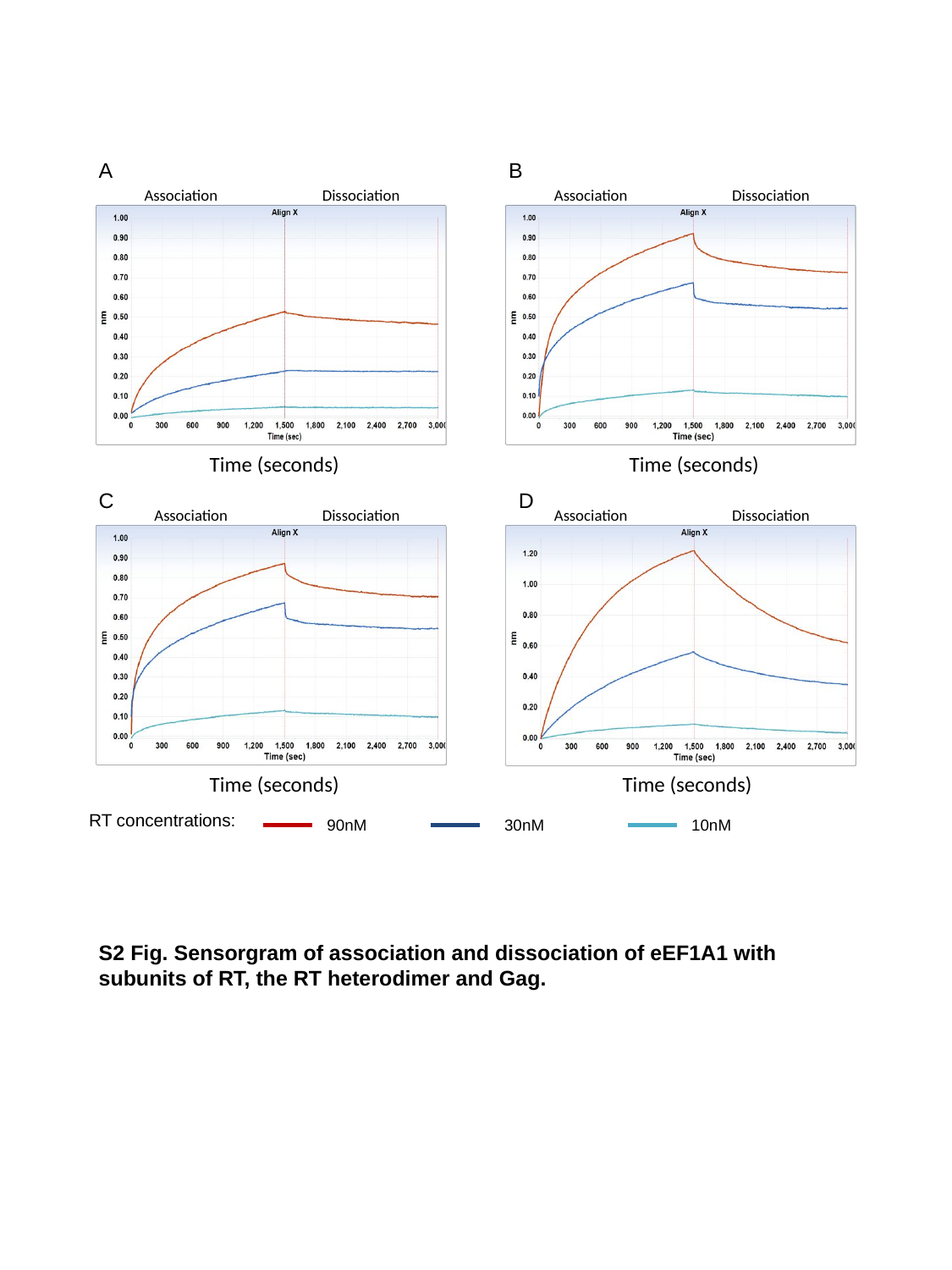

A
B
Association
Dissociation
Association
Dissociation
Time (seconds)
Time (seconds)
C
D
Association
Dissociation
Association
Dissociation
Time (seconds)
Time (seconds)
RT concentrations:
90nM
30nM
10nM
S2 Fig. Sensorgram of association and dissociation of eEF1A1 with subunits of RT, the RT heterodimer and Gag.
