# Supplementary material for: Specific Interaction between eEF1A and HIV RT Is Critical for HIV-1 Reverse Transcription and a Potential Anti-HIV Target
Source: PLoS Pathog. 2015 Dec 1;11(12):e1005289. doi: 10.1371/journal.ppat.1005289 (PMC4666417; doi:10.1371/journal.ppat.1005289)
Supplement: S3 Fig — FLAG-tagged RT51 was expressed in HEK293T cells by plasmid transfection. Cell lysates were prepared 24 h post-transfection and subjected to immunoprecipitation using an anti-FLAG antibody. The detection of eEF1A (A), eIF3A (B) and RT (C) in cell lysate (left column) and the co-immunoprecipitated eEF1A (right column, top panel) were detected by western blot. A lysate from mock-transfected cells was used as a control. (PPTX) [file ppat.1005289.s003.pptx]

## Slide 1
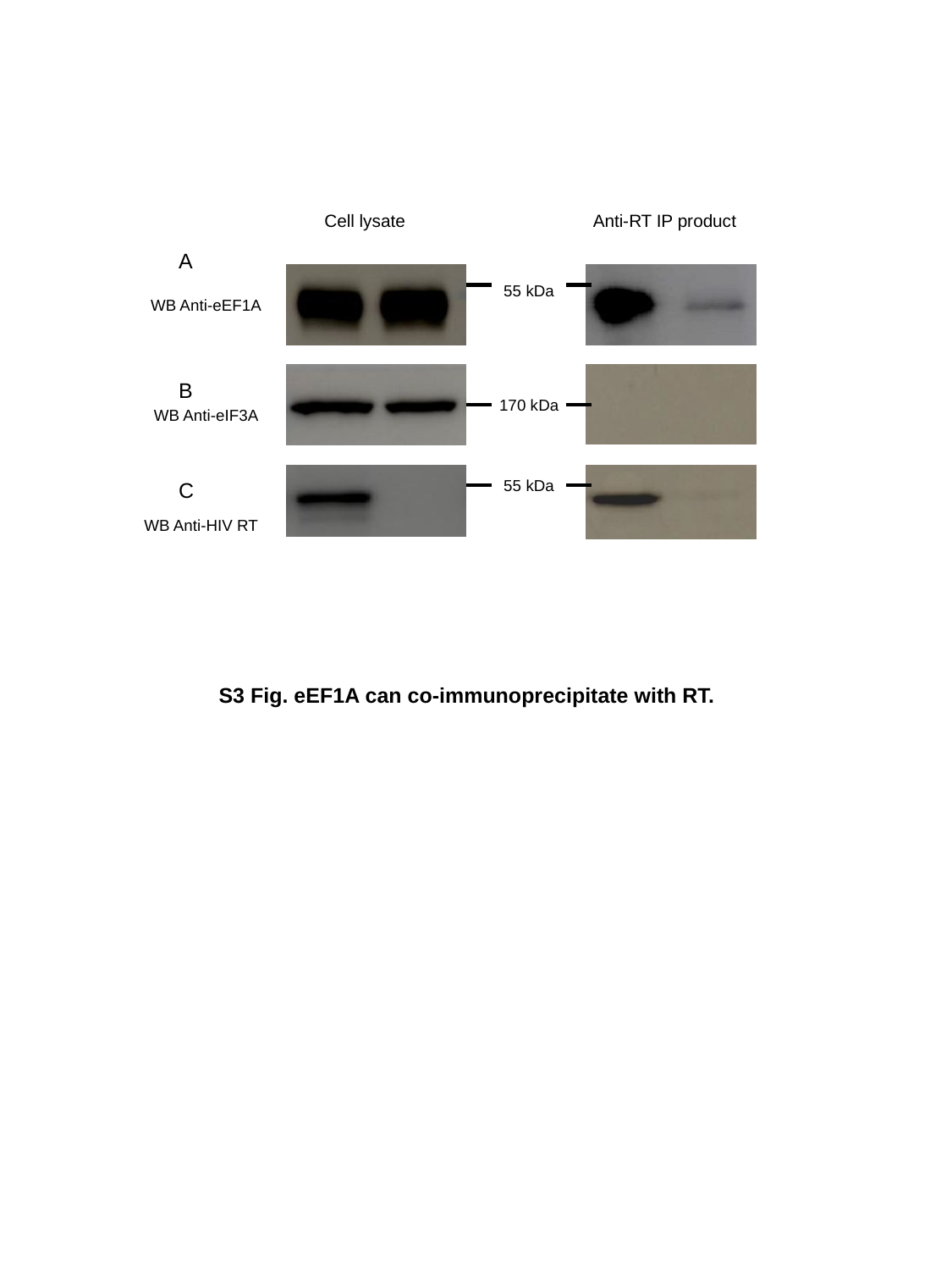

Cell lysate
Anti-RT IP product
A
55 kDa
WB Anti-eEF1A
B
170 kDa
WB Anti-eIF3A
55 kDa
C
WB Anti-HIV RT
S3 Fig. eEF1A can co-immunoprecipitate with RT.
