# Supplementary material for: Specific Interaction between eEF1A and HIV RT Is Critical for HIV-1 Reverse Transcription and a Potential Anti-HIV Target
Source: PLoS Pathog. 2015 Dec 1;11(12):e1005289. doi: 10.1371/journal.ppat.1005289 (PMC4666417; doi:10.1371/journal.ppat.1005289)
Supplement: S4 Fig — The W252 is located in the loop region just before the first alpha-helix of the thumb domain and is shown in sphere representation (red) within each subunit. (PPTX) [file ppat.1005289.s004.pptx]

## Slide 1
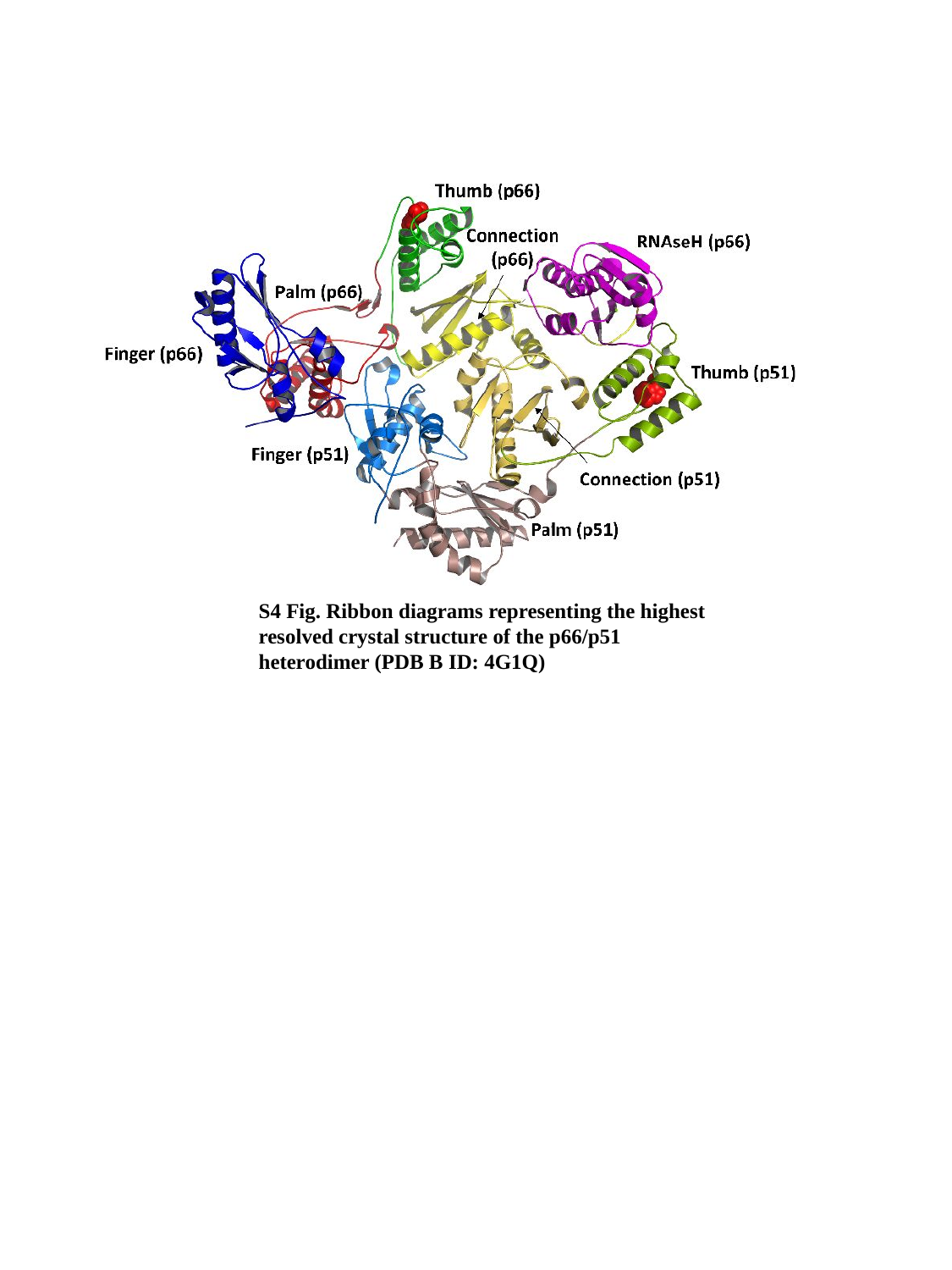

S4 Fig. Ribbon diagrams representing the highest resolved crystal structure of the p66/p51 heterodimer (PDB B ID: 4G1Q)
