# Supplementary material for: Specific Interaction between eEF1A and HIV RT Is Critical for HIV-1 Reverse Transcription and a Potential Anti-HIV Target
Source: PLoS Pathog. 2015 Dec 1;11(12):e1005289. doi: 10.1371/journal.ppat.1005289 (PMC4666417; doi:10.1371/journal.ppat.1005289)
Supplement: S5 Fig — Purified wild type RT p66 and mutant RT W252A, L303A mutants were analyzed using SDS-PAGE followed by Coomassie blue staining (A). In BLI assay, purified eEF1A was immobilized on the biosensors and incubated in solutions containing purified RT. The association maximums of 90 nM of wild type RT and mutants was compared (B). The data is presented as a mean value ± standard deviation from 3 independent experiments. *p<0.05. (PPTX) [file ppat.1005289.s005.pptx]

## Slide 1
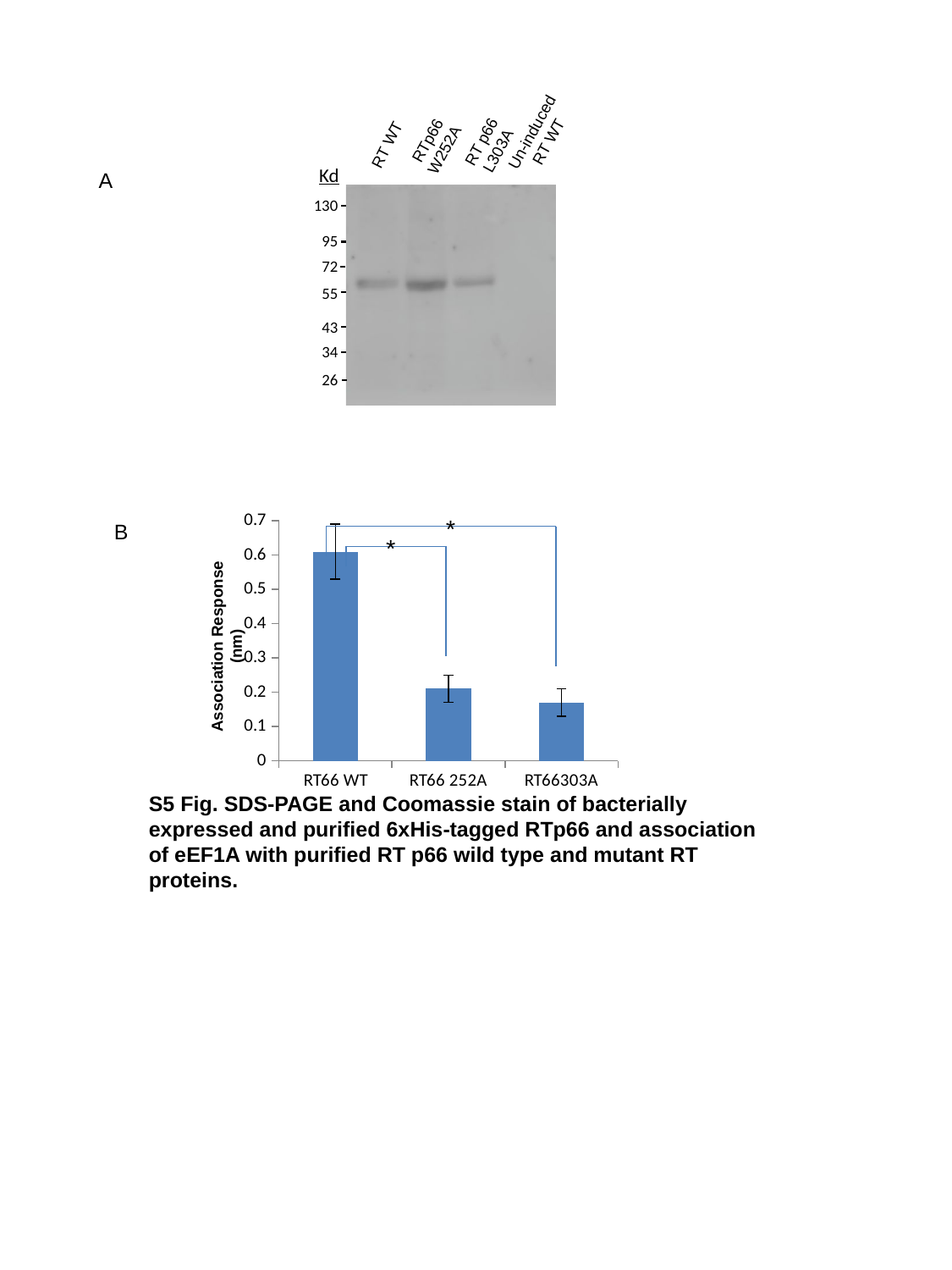

Un-induced
RT WT
RTp66 W252A
RT WT
RT p66 L303A
Kd
A
130
95
72
55
43
34
26
### Chart
| Category | |
|---|---|
| RT66 WT | 0.6100000000000004 |
| RT66 252A | 0.2100000000000001 |
| RT66303A | 0.17 |B
*
*
Association Response (nm)
S5 Fig. SDS-PAGE and Coomassie stain of bacterially expressed and purified 6xHis-tagged RTp66 and association of eEF1A with purified RT p66 wild type and mutant RT proteins.
