# Supplementary material for: Specific Interaction between eEF1A and HIV RT Is Critical for HIV-1 Reverse Transcription and a Potential Anti-HIV Target
Source: PLoS Pathog. 2015 Dec 1;11(12):e1005289. doi: 10.1371/journal.ppat.1005289 (PMC4666417; doi:10.1371/journal.ppat.1005289)
Supplement: S6 Fig — A leptin receptor deficient for STAT3 recruitment (red) is C-terminally to a bait protein (orange), in this case either RTp66 or RTp51. A flexible spacer (green) separates the fused proteins. A prey protein (purple) is N-terminally fused to a gp130 chain with four functional STAT3 recruitment sites. In the presence of ligand, interaction between the bait and prey leads to complementation of STAT3 signaling and activation of a reporter luciferase gene expressed by the rPAP1 promoter. (PPTX) [file ppat.1005289.s006.pptx]

## Slide 1
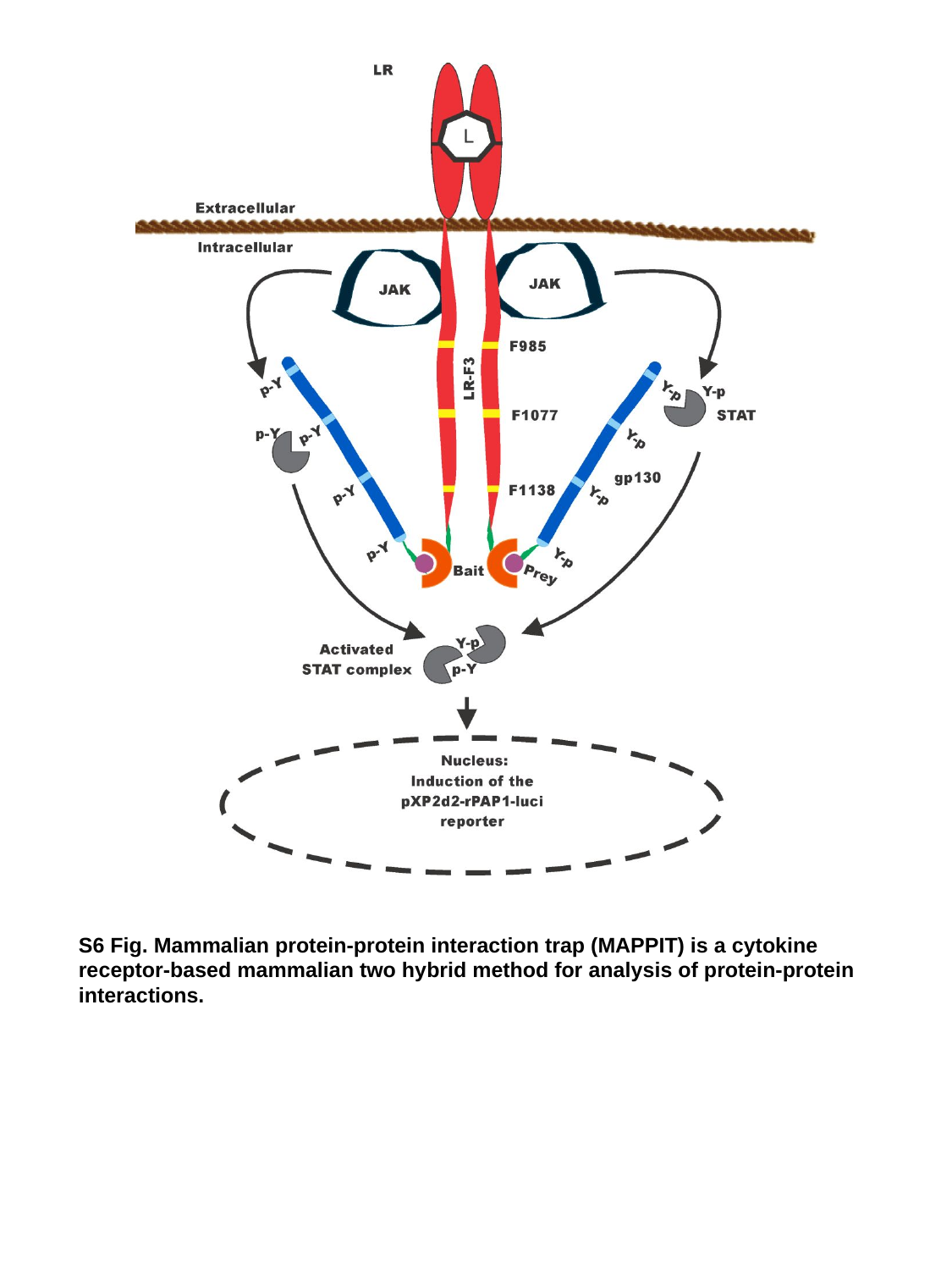

S6 Fig. Mammalian protein-protein interaction trap (MAPPIT) is a cytokine receptor-based mammalian two hybrid method for analysis of protein-protein interactions.
