# Supplementary material for: Specific Interaction between eEF1A and HIV RT Is Critical for HIV-1 Reverse Transcription and a Potential Anti-HIV Target
Source: PLoS Pathog. 2015 Dec 1;11(12):e1005289. doi: 10.1371/journal.ppat.1005289 (PMC4666417; doi:10.1371/journal.ppat.1005289)
Supplement: S7 Fig — The dimerization of RTp66 and RTp51 was investigated by MAPPIT. The pXP2d2-rPAP1-luciferase reporter plasmid was co-transfected with combinations of wild type or mutant, W252A (252A) and T253A (253A), RTp51 bait and RTp66 prey expression plasmids into HEK293T cells as shown. A bait construct having the myeloid differentiation primary response protein 88 (hMvD88) and a prey construct having SV40 large T antigen (SVT) were used as negative controls. Leptin (100 nM) was added to the cells at 20 h post-transfection and the cellular lysate was prepared 24 h later and used in luciferase assays. The data is presented as a mean value ± standard deviation from at least 3 independent experiments. (PPTX) [file ppat.1005289.s007.pptx]

## Slide 1
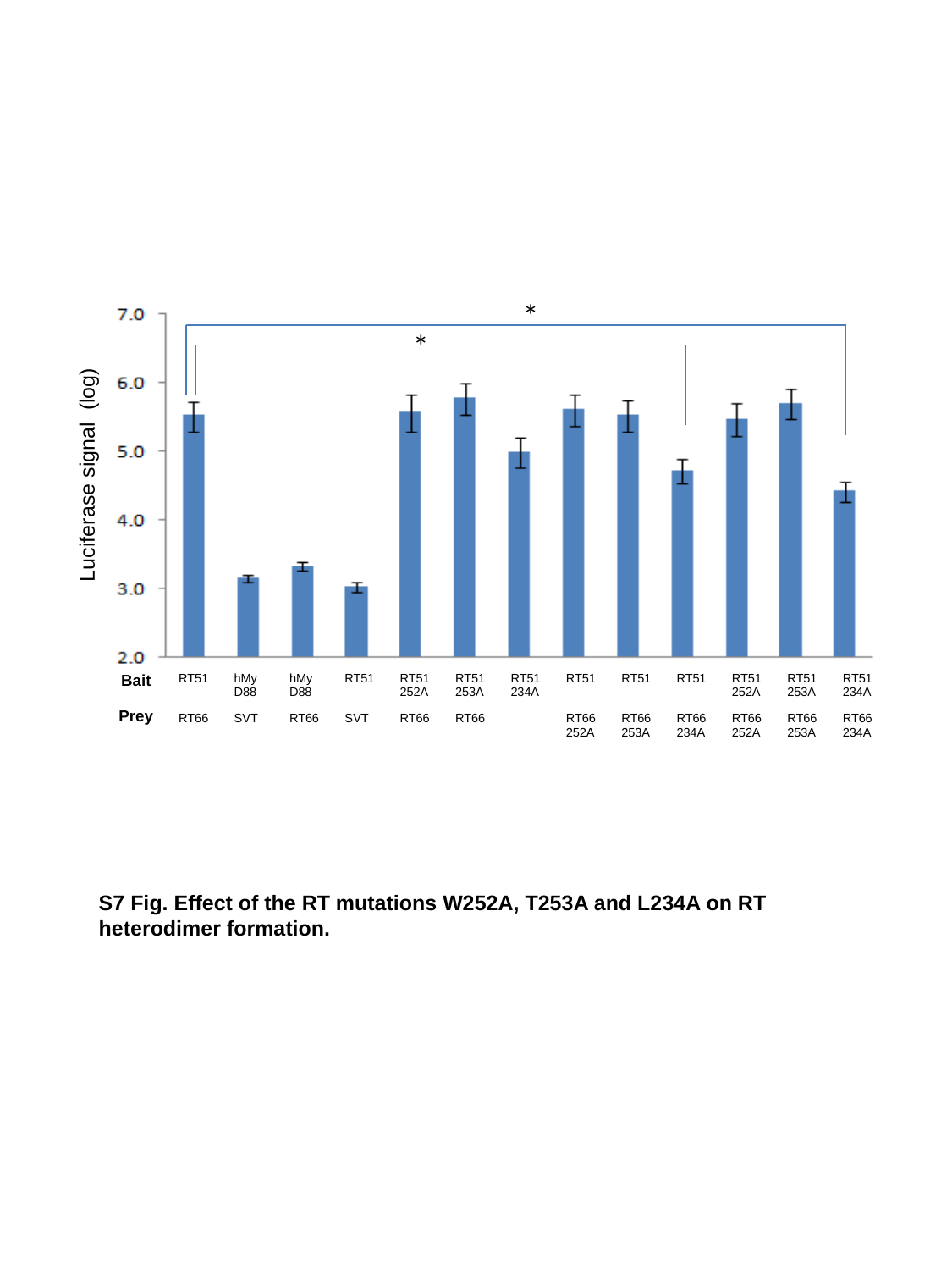

*
*
Luciferase signal (log)
Bait
| RT51 | hMy D88 | hMy D88 | RT51 | RT51252A | RT51253A | RT51 234A | RT51 | RT51 | RT51 | RT51252A | RT51253A | RT51 234A |
| --- | --- | --- | --- | --- | --- | --- | --- | --- | --- | --- | --- | --- |
| RT66 | SVT | RT66 | SVT | RT66 | RT66 | | RT66252A | RT66253A | RT66234A | RT66252A | RT66253A | RT66 234A |
Prey
S7 Fig. Effect of the RT mutations W252A, T253A and L234A on RT heterodimer formation.
