# Supplementary material for: Specific Interaction between eEF1A and HIV RT Is Critical for HIV-1 Reverse Transcription and a Potential Anti-HIV Target
Source: PLoS Pathog. 2015 Dec 1;11(12):e1005289. doi: 10.1371/journal.ppat.1005289 (PMC4666417; doi:10.1371/journal.ppat.1005289)
Supplement: S8 Fig — (A) The plasmid pCMV-Gluc2 was transfected into Jurkat cells for luciferase expression and Did B or CHX were added at concentrations as indicated. The levels of luciferase in culture supernatant were measured 24 h after treatment. (B) Jurkat cells were incubated with concentrations of Did B and CHX as indicated for 24 h at 37°C and then incubated with CellTiter 96 AQueous One Solution Cell Proliferation solution for 2 h at 37°C. The absorbance was measured at 490nm in a 96-well plate reader. The data is presented as a mean value ± standard deviation from at least 3 independent experiments. *p < 0.05 (PPTX) [file ppat.1005289.s008.pptx]

## Slide 1
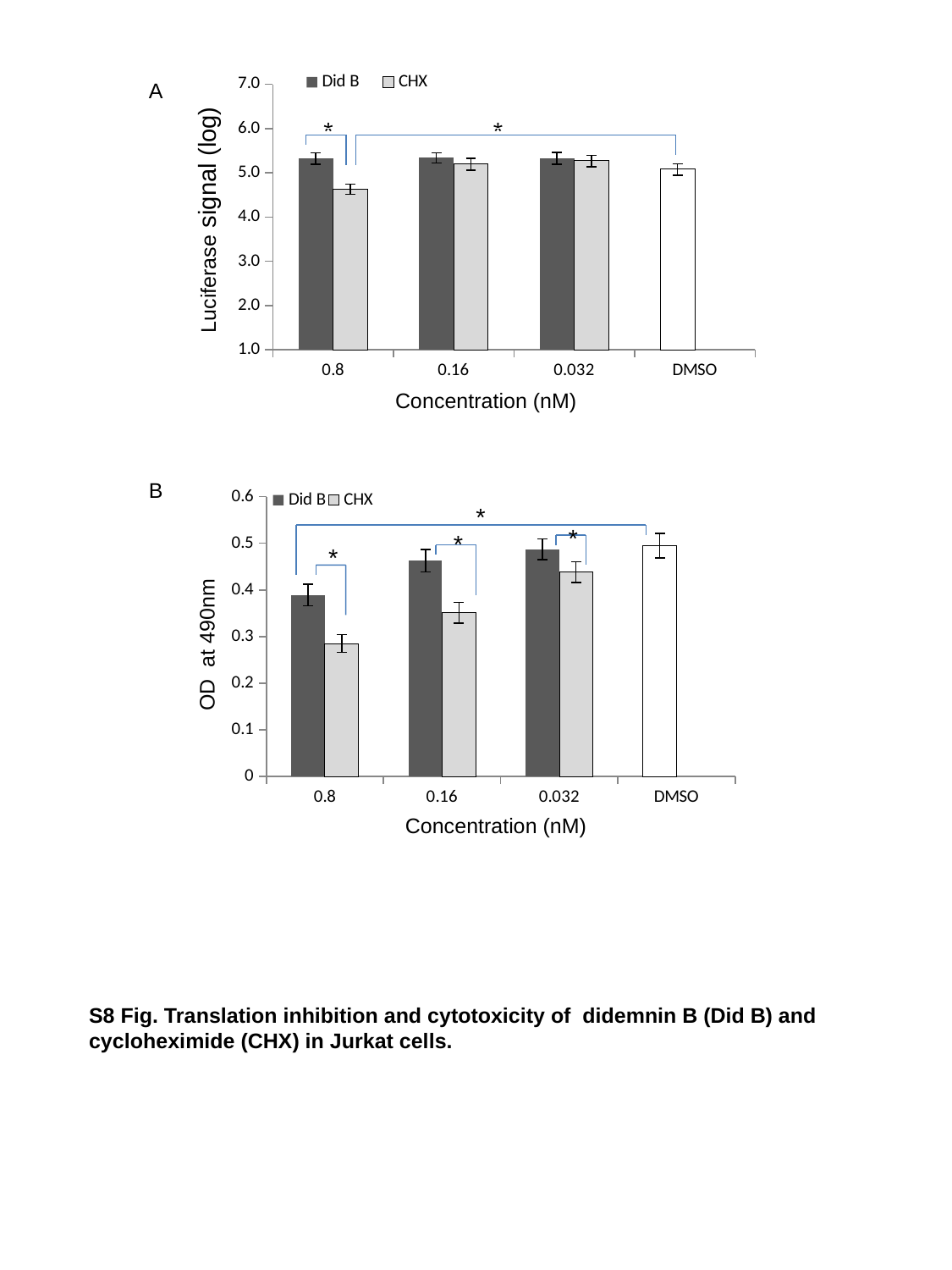

### Chart
| Category | Did B | CHX |
|---|---|---|
| 0.8 | 5.329772004747817 | 4.636658371116684 |
| 0.16 | 5.344822424806217 | 5.198854948221696 |
| 0.032 | 5.3331186583197345 | 5.276024992238583 |
| DMSO | 5.08350620310329 | None |A
*
*
Luciferase signal (log)
Concentration (nM)
B
### Chart
| Category | Did B | CHX |
|---|---|---|
| 0.8 | 0.38900000000000073 | 0.2850000000000003 |
| 0.16 | 0.463 | 0.3510000000000003 |
| 0.032 | 0.4870000000000003 | 0.43800000000000067 |
| DMSO | 0.4950000000000004 | None |*
*
*
*
OD at 490nm
Concentration (nM)
S8 Fig. Translation inhibition and cytotoxicity of didemnin B (Did B) and cycloheximide (CHX) in Jurkat cells.
