# Supplementary material for: Specific Interaction between eEF1A and HIV RT Is Critical for HIV-1 Reverse Transcription and a Potential Anti-HIV Target
Source: PLoS Pathog. 2015 Dec 1;11(12):e1005289. doi: 10.1371/journal.ppat.1005289 (PMC4666417; doi:10.1371/journal.ppat.1005289)
Supplement: S9 Fig — (A) The copy number of COXII in DNA samples extracted from Did B and CHX treated cells. Jurkat cells were incubated with various concentrations of Did B and CHX followed by HIV-1 infection. The cytoplasmic nucleic acids were extracted from cells and COXII levels were measured by qPCR. (B) In vitro RT activity in the presence of Did B, CHX and nevirapine (NVP). RT (0.5 ng) was incubated with concentrations of Did B, CHX and NVP using the Roche Reverse Transcriptase Assay (colorimetric). The data is presented as a mean value ± standard deviation from at least 3 independent experiments. (PPTX) [file ppat.1005289.s009.pptx]

## Slide 1
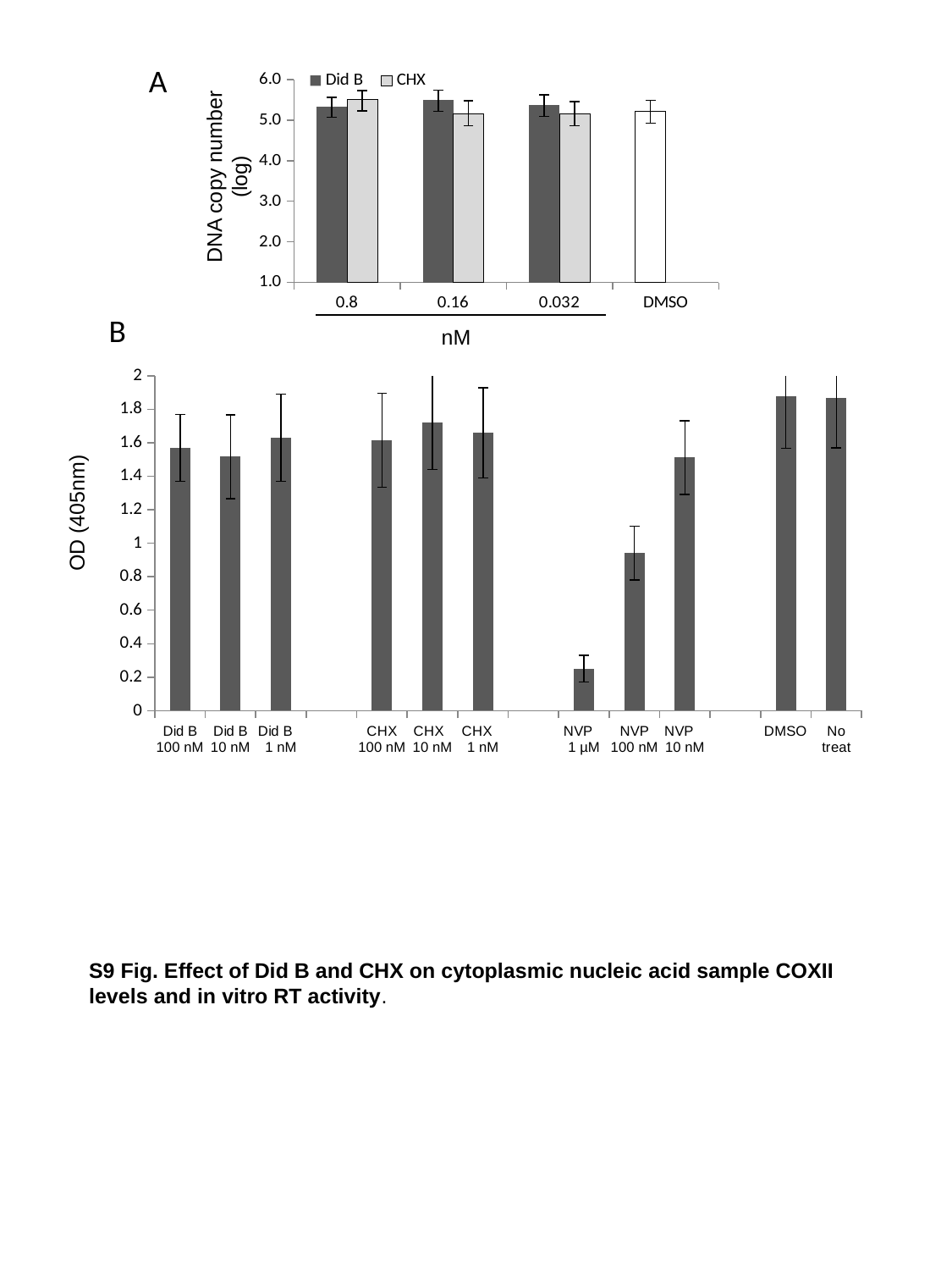

A
### Chart
| Category | Did B | CHX |
|---|---|---|
| 0.8 | 5.33069982642125 | 5.507175573873809 |
| 0.16 | 5.4934078567580675 | 5.1576229589939855 |
| 0.032 | 5.371804613582175 | 5.1634953192317665 |
| DMSO | 5.222503172384223 | None |DNA copy number (log)
nM
B
### Chart
| Category | |
|---|---|
| Did B 100 nM | 1.569 |
| Did B 10 nM | 1.516 |
| Did B 1 nM | 1.629 |
| | None |
| CHX 100 nM | 1.615 |
| CHX 10 nM | 1.720999999999997 |
| CHX 1 nM | 1.659 |
| | None |
| NVP 1 µM | 0.252 |
| NVP 100 nM | 0.941 |
| NVP 10 nM | 1.510999999999997 |
| | None |
| DMSO | 1.875999999999997 |
| No treat | 1.868 |OD (405nm)
S9 Fig. Effect of Did B and CHX on cytoplasmic nucleic acid sample COXII levels and in vitro RT activity.
